# Disruption of placental ACKR3 impairs growth and hematopoietic development of offspring

**DOI:** 10.1242/dev.202333

**Published:** 2024-02-23

**Authors:** Ayumi Fukuoka, Gillian J. Wilson, Elise Pitmon, Lily Koumbas Foley, Hanna Johnsson, Marieke Pingen, Gerard J. Graham

**Affiliations:** Chemokine Research Group, School of Infection and Immunity, College of Medical, Veterinary and Life Sciences, University of Glasgow, 120 University Place, Glasgow G12 8TA, UK

**Keywords:** Atypical chemokine receptor, Haematopoietic stem cells, Mouse, Immune responses

## Abstract

ACKR3 scavenges and degrades the stem cell recruiting chemokine CXCL12, which is essential for proper embryonic and, in particular, haematopoietic development. Here, we demonstrate strong expression of ACKR3 on trophoblasts. Using a maternally administered pharmacological blocker and Cre-mediated genetic approaches, we demonstrate that trophoblast ACKR3 is essential for preventing movement of CXCL12 from the mother to the embryo, with elevated plasma CXCL12 levels being detected in embryos from ACKR3-blocker-treated mothers. Mice born to mothers treated with the blocker are lighter and shorter than those born to vehicle-treated mothers and, in addition, display profound anaemia associated with a markedly reduced bone marrow haematopoietic stem cell population. Importantly, although the haematopoietic abnormalities are corrected as mice age, our studies reveal a postnatal window during which offspring of ACKR3-blocker-treated mice are unable to mount effective inflammatory responses to inflammatory/infectious stimuli. Overall, these data demonstrate that ACKR3 is essential for preventing CXCL12 transfer from mother to embryo and for ensuring properly regulated CXCL12 control over the development of the haematopoietic system.

## Teaser

**Highlighted Article:** Placental ACKR3 plays an essential role in compartmentalising CXCL12 activity on the maternal side of the placenta: blockade of ACKR3 leads to severe developmental abnormalities and disrupted haematopoiesis and immune response in offspring.

## INTRODUCTION

The migration of leukocytes is controlled predominantly by a family of cytokines, called chemokines, and their receptors (Bachelerie et al., 2014a; Sokol and Luster, 2015). The chemokine family is defined on the basis of a conserved cysteine motif and divided into four subfamilies according to the specific nature of this motif (CC, CXC, XC and CX3C families) (Zlotnik and Yoshie, 2000).

The chemokine family emerged in early vertebrates (Nomiyama et al., 2011; Zlotnik et al., 2006) and the primordial chemokine is believed to be CXCL12 (Karpova and Bonig, 2015; Lewellis and Knaut, 2012; Zlotnik et al., 2006). Mice with a homozygous null deletion in either CXCL12 or its receptor CXCR4 (Nagasawa et al., 1996; Tachibana et al., 1998; Zou et al., 1998) die perinatally, and analyses indicate that they have severely depleted bone marrow (BM) haematopoiesis as well as numerous other abnormalities including disrupted vascular development and alterations to cortical interneuron development (Stumm and Höllt, 2007). Further analysis using zebrafish has shown that primordial germ cells also express CXCR4 and that animals with homozygous null deletion in CXCL12 or CXCR4 are effectively sterile (Doitsidou et al., 2002; Molyneaux et al., 2003). Thus, the pairing of CXCL12 and CXCR4 is essential for stem cell migration and the development of numerous tissue systems within the embryo, and interfering with this axis has profound implications for offspring.

In addition to the classical chemokine receptors, there exists a subfamily of receptors called atypical chemokine receptors (ACKRs) (Bachelerie et al., 2014b; Bonecchi and Graham, 2016; Nibbs and Graham, 2013). These are 7-transmembrane spanning receptors, but they lack the typical signalling capabilities of the other chemokine receptors. There are currently four members of this family, labelled ACKR1-4. Functionally, with the exception of ACKR1, all ACKRs actively internalise and scavenge their ligands through lysosomal-driven degradation (Bonecchi et al., 2004; Bryce et al., 2016; Naumann et al., 2010; Weber et al., 2004). They are therefore involved in removing chemokines in specific tissue contexts and in sculpting chemokine gradients. In terms of ligands, ACKR3 (also known as CXCR7) predominantly binds, internalises and scavenges the primordial chemokine CXCL12. ACKR3 knockout is associated with perinatal lethality linked to disrupted cardiac development (Gerrits et al., 2008; Sierro et al., 2007; Yu et al., 2011). ACKR3 therefore presents itself as an important regulator of the *in vivo* activities of the chemokine-receptor pairing of CXCL12 and CXCR4.

We have previously demonstrated that ACKR2 is expressed on trophoblasts in the junctional zone and that its primary function here is to ensure that the mother can mount systemic chemokine-driven responses without these ligands entering the fetal circulation and interfering with cell migration within the embryo (Lee et al., 2019). We refer to this as ‘chemokine compartmentalisation’ and it involves trophoblast ACKR2 scavenging its ligands on the maternal face of the placenta, thereby preventing their transplacental entry into the embryonic circulation.

Here, we demonstrate that ACKR3 is expressed in syncytiotrophoblasts and is essential for compartmentalisation of CXCL12. As ACKR3 deletion is perinatally lethal in mice, and as the most common trophoblast-specific Cre-driver (Cyp19-Cre) is associated with extensive mosaicism and occasional lack of activity in placentas (Anamthathmakula et al., 2023), we have performed these studies using a well-characterised and selective pharmacological blocker of ACKR3 (Zabel et al., 2009). Crucially, to complement the pharmacological blocker data, we have replicated key findings using Cyp19-Cre deletion of ACKR3. Overall, our data show that maternally delivered pharmacological blockade, and trophoblast-specific deletion, of ACKR3 is associated with increased levels of maternally derived CXCL12 in embryonic plasma. This impacts general embryonic development and severely blunts the seeding of BM by haematopoietic stem cells (HSC) and haematopoietic progenitor cells (HPC). Pups born to mothers exposed to ACKR3 blockade are smaller than those born to control mothers. Furthermore, they display profound anaemia and haematopoietic insufficiency, which is associated with an inability to mount protective inflammatory responses to bacterial stimuli. This study, therefore, demonstrates an essential role for placental ACKR3 in compartmentalising CXCL12 activity on the maternal side of the placenta, thereby ensuring proper haematopoiesis and immune response in offspring.

## RESULTS

### ACKR3 is expressed in placental trophoblasts

**Fig. 1.**
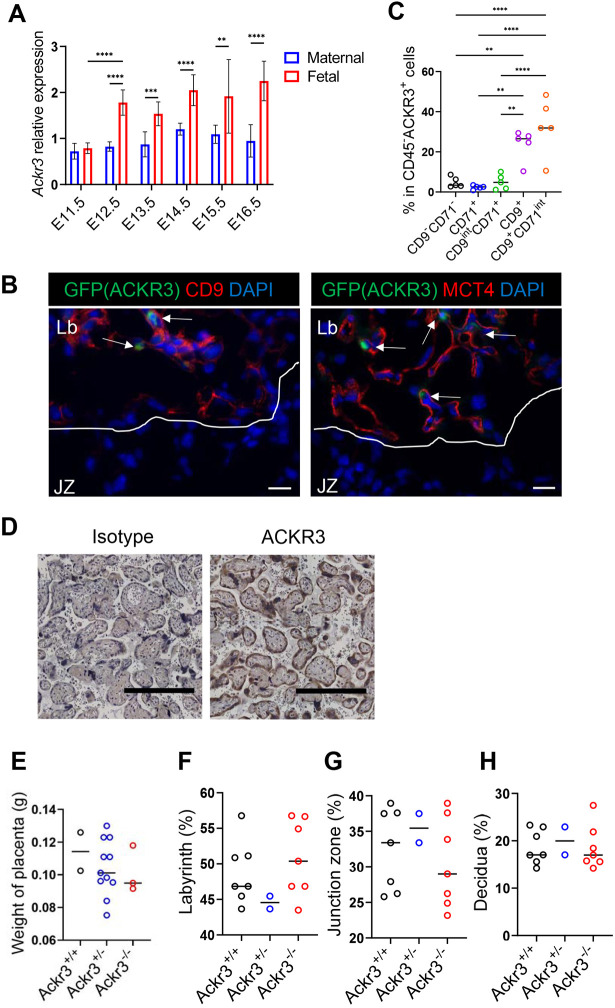
**ACKR3 is expressed in the placenta.** (A) Relative expression levels of *Ackr3* mRNA in mouse placentas and maternal decidua between E11.5 and E16.5. *n*=6 at E11.5, *n*=10 at E12.5, *n*=10 at E13.5, *n*=9 at E14.5, *n*=6 at E15.5 and *n*=7 at E16.5. (B) Representative images of placentas of ACKR3 reporter mice at E15.5. Sections were stained with syncytiotrophoblast (SynT) markers. Arrows show ACKR3^+^ cells. Left image: red, CD9 (SynT-I and -II); blue, DAPI; green, ACKR3. Right image: red, Mct4 (SynT-II); blue, DAPI; green, ACKR3. Lb, labyrinth; JZ, junction zone. Scale bars: 20 μm. *n*=5. (C) Representative flow cytometry plots of trophoblast cells in ACKR3 reporter mice. The graph shows percentages of CD9^−^CD71^−^, CD71^+^, CD9^int^CD71^+^, CD9^+^ and CD9^+^CD71^int^ cells in ACKR3^+^ cells in fetal side of placentas. *n*=5. (D) Representative images of immunohistochemistry of human placentas. Sections were stained with an anti-human ACKR3 antibody and counterstained with Haematoxylin. Scale bars: 0.5 µm. *n*=3. (E) Weight of placentas at E15.5. Wild-type, *n*=2; Ackr3^GFP/+^, *n*=11; Ackr3^GFP/GFP^, *n*=3. (F-H) Histological analysis of placentas at E15.5. The graphs show proportions of labyrinth (F), junction zone (G) and decidua (H). Wild-type, *n*=7; Ackr3^GFP/+^, *n*=2; Ackr3^GFP/GFP^, *n*=7. Data are representative of at least two independent experiments. Mean±s.d. ****P<0.001, ***P<0.005, **P<0.01 [two-way ANOVA with Bonferroni's post-test (A) or one-way ANOVA with Tukey's post-test (C,E-H)]. See also Figs S1 and S2.

We used quantitative real-time PCR (qRT-PCR) to examine ACKR3 expression in placenta and maternal decidua from wild-type (WT) pregnant mice at various times post conception. ACKR3 expression was detected in maternal decidua and did not alter across all time points (Fig. 1A). However, increased expression was apparent in the placenta starting at embryonic day (E)12.5 and continued at a similar level until E16.5. Higher expression in the placenta, compared with the maternal decidua, was also apparent at E18.5, at which time expression in the placenta was also higher than in any of the other embryonic tissues tested (Fig. S1A). Using *Ackr3^GFP^* reporter mice, along with co-staining for syncytiotrophoblast markers CD9 and MCT4 (also known as Slc16a3), expression of ACKR3 was localised to the labyrinth region of the mouse placenta: specifically it was expressed on syncytiotrophoblasts, which form a barrier between maternal blood and embryonic tissues (Fig. 1B; Fig. S1B). Furthermore, flow cytometric analysis indicated clear association of ACKR3^+^ placental cells with the syncytiotrophoblast markers CD9 and CD71 (Tfrc), but lack of association with CD45 (Ptprc; haematopoietic cells), CD31 (Pecam1; endothelial cells), CD90 (Thy1; fibroblasts) and EpCAM (epithelial cells) (Fig. 1C; Fig. S1C,D). In addition, immunostaining of human placenta localises ACKR3 expression to syncytiotrophoblasts (Fig. 1D; Fig. S2A), confirming that trophoblast expression of ACKR3 is evolutionarily maintained within mammals. Thus, ACKR3 is expressed predominantly in the placenta and its expression is restricted to syncytiotrophoblasts.

To investigate whether deletion of ACKR3 affects placental development or structure, we carried out gross analysis of placentas taken from WT, heterozygous (*Ackr3^GFP/+^*) and homozygous (*Ackr3^GFP/GFP^*) litter mates. This gross analysis did not reveal any significant differences in overall placental structure across the different genotypes (Fig. S2B). To analyse placental defects more precisely, we compared placental weight between the genotypes (Fig. 1E) and the percentages of placental cross-section taken up by the decidua, labyrinth and junctional zones (Fig. 1F-H). None of these parameters were significantly different between the genotypes. Therefore, the absence of ACKR3 appears to have no detectable gross impact on the placental development.

### ACKR3 regulates CXCL12 passage into the embryo

**Fig. 2.**
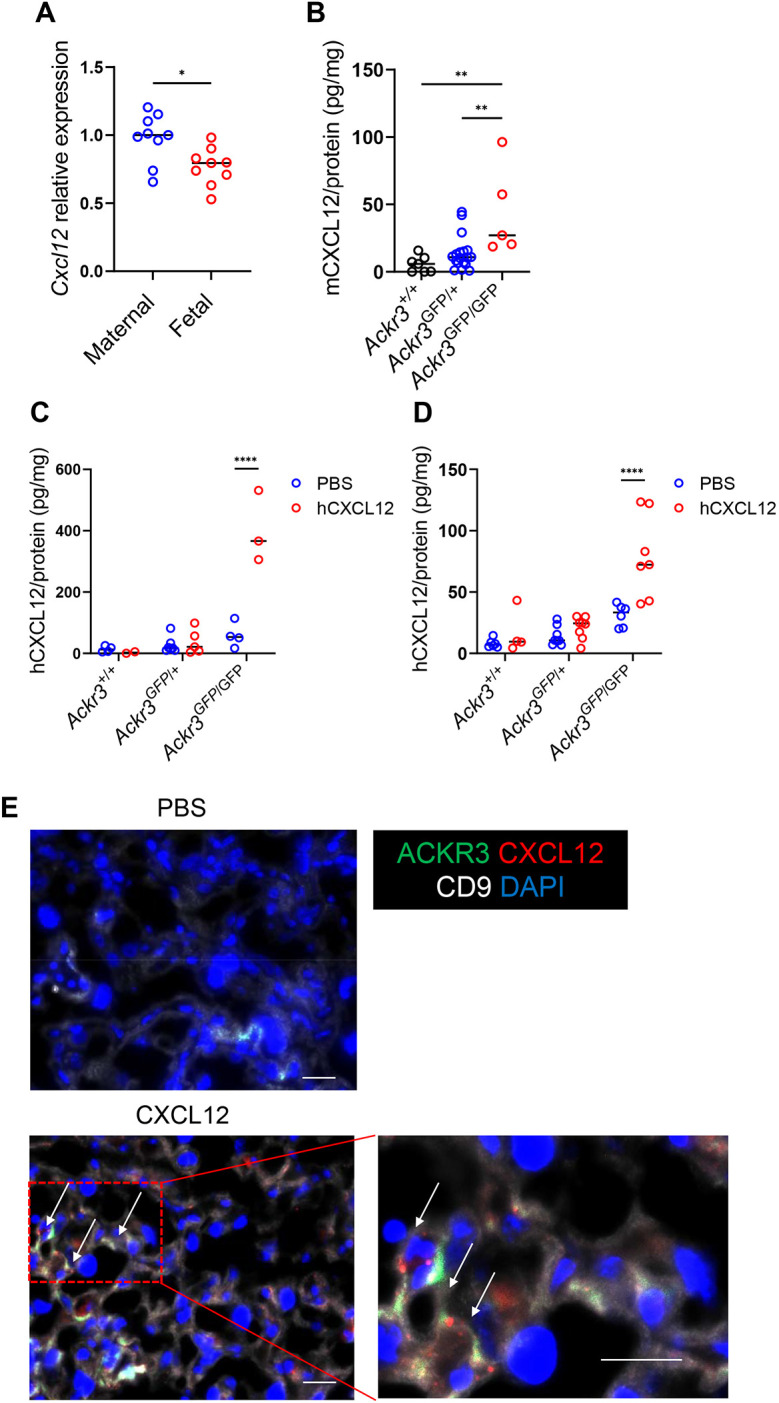
**Placental ACKR3 prevents maternal CXCL12 entry into the embryonic circulation.** (A) Relative expression levels of *Cxcl12* mRNA in maternal decidua (maternal) and placenta (fetal) side of isolated WT placentas at E15.5. *n*=9. (B) Mouse CXCL12 protein levels in plasma of embryos at E13.5 normalised to mg of total protein. Wild-type, *n*=6; Ackr3^GFP/+^, *n*=18; Ackr3^GFP/GFP^, *n*=5. (C,D) CXCL12 levels, measured by ELISA, in plasma of embryos at E12.5 (C) and E15.5 (D) after hCXCL12 injection and normalised to mg of protein. Wild-type, *n*=6 (C) or 8 (D); Ackr3^GFP/+^, *n*=10 (C) or 11 (D); Ackr3^GFP/GFP^ *n*=7 (C) or 13 (D). (E) *Ackr3*^GFP/+^ placentas were obtained after Alexa647-conjugated CXCL12 and control sections were stained with anti-CD9 antibody. Arrows show ACKR3^+^ cells scavenging CXCL12. Green, ACKR3; white, CD9; red, CXCL12; blue, DAPI. Scale bars: 20 μm. *n*=4. Data are representative of at least two independent experiments (A,C,D) or were pooled from two independent experiments (B). Graphs show mean. ****P<0.001, **P<0.01, *P<0.05 [two-tailed unpaired Student's *t*-test (A), one-way ANOVA with Tukey's post-test (B) or two-way ANOVA with Bonferroni's post-test (C,D)].

Next, we examined whether ACKR3 can compartmentalise chemokine function on the placenta. CXCL12 is detectable, at the transcript level, in both maternal decidua and placenta (Fig. 2A). To determine roles for placental ACKR3 in limiting CXCL12 movement from maternal circulation to the embryo, we crossed heterozygous male and female mice (note that ACKR3 homozygosity is perinatally lethal; Gerrits et al., 2008; Sierro et al., 2007; Yu et al., 2011) to give rise to WT, heterozygous and knockout embryos within each litter. We used an ELISA which is specific for CXCL12 to assess alterations in CXCL12 levels in plasma of *Ackr3*-deficient embryos. Plasma CXCL12 levels at E13.5 were significantly higher in *Ackr3*-deficient embryos compared with WT embryos (Fig. 2B). With an approximate plasma protein concentration of 80 mg/ml, mean CXCL12 levels detected in the *Ackr3*-deficient embryonic plasma were equivalent to 2 ng/ml, placing them within the bioactive range (Wu et al., 2010). To complement these analyses, and notwithstanding the limitations of the Cyp19-Cre driver line (Anamthathmakula et al., 2023), we also measured CXCL12 levels in the plasma of embryos which were positive for both Cyp19-Cre and the ACKR3 floxed allele. The data show significantly higher levels of CXCL12 in the plasma from Cre^+/−^ Ackr3^fl/fl^ mice compared with Cre^−/−^ Ackr3^fl/fl^ mice (Fig. S3), thus substantiating the data from the heterozygote crosses. Overall, these data indicate that placental ACKR3 regulates CXCL12 levels in embryos.

To examine the ability of ACKR3 to prevent CXCL12 passage from the mother to the embryo, we next injected *Ackr3*^GFP/+^ pregnant mothers crossed with *Ackr3*^GFP/+^ males with CXCL12. ELISA was used to measure chemokine movement from maternal plasma to fetal plasma. CXCL12 was readily detectable at both E12.5 and E15.5 in knockout, but not WT, embryonic plasma (Fig. 2C,D). Again, assuming an approximate total plasma protein concentration of 80 mg/ml, the peak levels of CXCL12 at E12.5 and E15.5 are equivalent to approximately to 5 ng/ml and 30 ng/ml respectively. Both concentrations are, therefore, clearly within the bioactive range.

Finally, to examine whether ACKR3-expressing trophoblasts take up CXCL12, *Ackr3*^GFP/+^ placentas were stained with a syncytiotrophoblast marker after injection of AF647-labelled CXCL12. AF647-CXCL12 was clearly seen to localize with ACKR3-expressing trophoblasts and to be included in structures resembling endosomes (Fig. 2E).

These data demonstrate that ACKR3 on trophoblasts regulates CXCL12 passage from the placenta and the maternal circulation into the embryo.

### Pharmacological blockade of ACKR3 substantially compromises embryonic growth

**Fig. 3.**
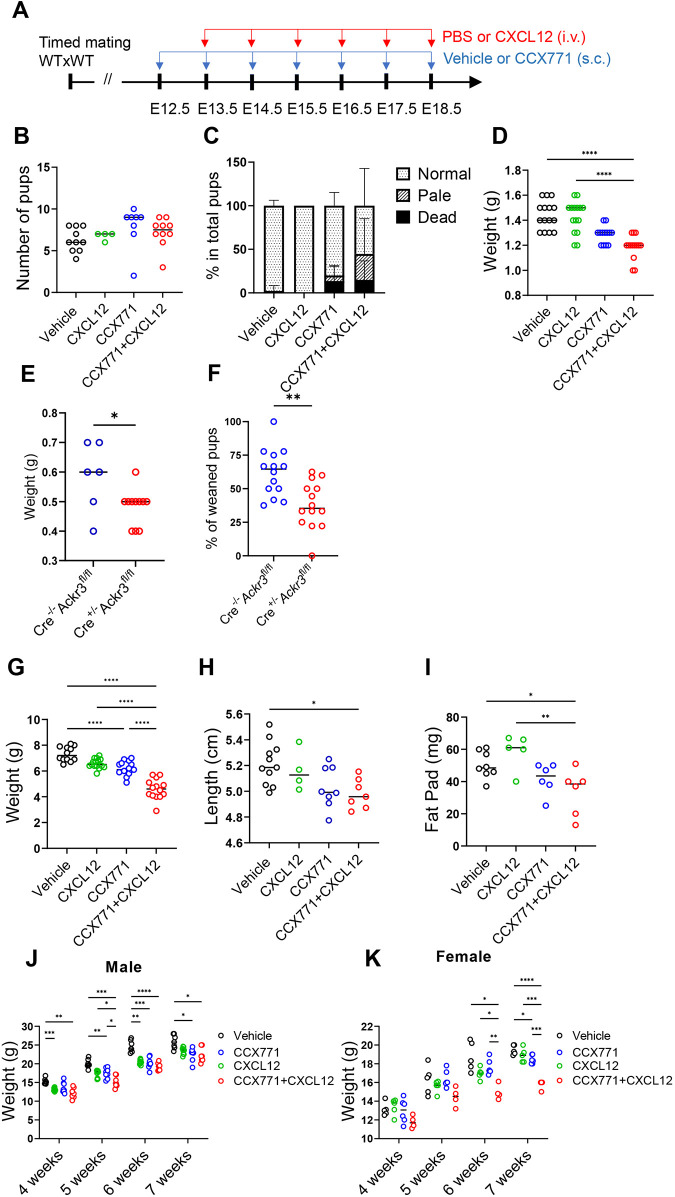
**Blockade of ACKR3 during pregnancy affects embryonic development.** (A) Experimental design. Wild-type (WT) pregnant mice were subcutaneously (s.c.) injected with vehicle or CCX771 between E12.5 and E18.5. Additionally, the mice were intravenously (i.v.) injected with PBS or CXCL12 between E13.5 and E18.5. (B-D) Analysis of neonates born to the injected pregnant mice: number of pups (B); the percentages of normal, pale and dead pups at postnatal day 0 (P0) (C); weight of P0 pups (D). Vehicle, *n*=10 (B), 10 (C) or 15 (D); CXCL12 group, *n*=4 (B,C) or 13 (D); CCX771 group, *n*=8 (B,C) or 12 (D); CCX771+CXCL12 group, *n*=10 (B-D). (E) Weight of E15.5 embryos. Cre^−/−^ Ackr3^fl/fl^ group, *n*=6; Cre^+/−^ Ackr3^fl/fl^ group, *n*=12. (F) Percentage of weaned pups from six Cre^+/−^ Ackr3^fl/fl^ mother mice mated with Cre^−/−^ Ackr3^fl/fl^ males. Cre^−/−^ Ackr3^fl/fl^ group, *n*=14; Cre^+/−^ Ackr3^fl/fl^ group, *n*=14. (G-I) Analysis of 2-week-old pups: weight of 2-week-old pups (E); length of pups (F); weight of fat pads (G). Vehicle, *n*=12 (E), 11 (F) or 8 (G); CXCL12 group, *n*=15 (E), 4 (F) or 5 (G); CCX771 group, *n*=15 (E), 8 (F) or 6 (G); CCX771+CXCL12 group, *n*=14 (E), 7 (H) or 6 (G). (J,K) Weight of males (J) and females (K) between 4 and 7 weeks of age. Data were pooled from two independent experiments. Vehicle group, *n*=7 (H) or 5 (I); CXCL12 group, *n*=9 (H) or 5 (I); CCX771 group, *n*=8 (H) or 6 (I); CCX771+CXCL12 group, *n*=7 (H) or 4 (I). Mean±s.d. ****P<0.001, ***P<0.005, **P<0.01, *P<0.05 [one-way ANOVA with Tukey's post-test (B,D,E-G), two-tailed unpaired Student's *t*-test (E,F) or two-way ANOVA with Bonferroni's post-test (J,K)].

As ACKR3 deletion is perinatally lethal, and given the limitations of the Cre-based approach, we opted to examine the developmental relevance of placental ACKR3 using a well-characterised pharmacological blocker, CCX771 (Zabel et al., 2009) (Fig. 3A). This blocker was administered to pregnant mice with the aim of inhibiting ACKR3 on trophoblasts which are fully exposed to maternal blood. Injection of WT mothers with CCX771 did not significantly affect the total number of newborns within each litter, but was associated with an increase in the numbers of dead newborns per litter (Fig. 3B,C). In addition, we observed a number of pale-looking neonates, particularly when CCX771 was co-administered with CXCL12 to the mother (Fig. 3C). Embryos in CCX771-treated mothers showed high CXCL12 levels in the blood compared with control embryos, indicating that maternal CXCL12 entered the embryonic circulation following inhibition of ACKR3 (Fig. S4A). Strikingly, CCX771 administration was also associated with a marked and significant reduction in the weight of neonates, especially when combined with maternally administered CXCL12 (Fig. 3D). These data were confirmed using the Cyp19-Cre approach which, when bred onto an ACKR3^fl/fl^ background, yielded smaller and paler embryos and significantly reduced numbers of offspring (Fig. 3E,F; Fig. S4B). Analysis at 2 weeks of age of pups born to CCX771-treated mothers showed that this reduction in body weight was also apparent at this time (Fig. 3G) alongside a reduction in the length of the pups (Fig. 3H; Fig. S5A) and in the weight of the fat pads (Fig. 3I). Analysis of mouse weight up to 7 weeks showed that, particularly for female mice, pups born to mothers receiving both CCX771 and CXCL12 never recovered the weight disadvantage with which they were born (Fig. 3J,K).

Importantly, mass spectrometry analysis of embryonic plasma revealed that CCX771 administered to the mother is frequently undetectable in the embryo and, where levels are detected, they were well below the IC_50_ for this blocker (18 ng/ml). Additionally, where low level CCX771 was detected in embryos, this did not positively correlate with the decreased HSC population described below (Fig. S5B). This demonstrates that CCX771 does not cross the placenta in biologically significant levels and that, where low levels are detected in embryonic circulation, they cannot account for the phenotypes reported. Overall, these data indicate that the ACKR3-blocking activity of CCX771 is restricted to the mother and most likely to trophoblastic cells.

Overall, these data demonstrate that trophoblastic ACKR3 blockade, particularly in combination with maternally administered CXCL12, leads to embryonic compromise associated with reduced body weight, which is maintained until maturity.

### ACKR3 blockade substantially impairs haematopoietic development

**Fig. 4.**
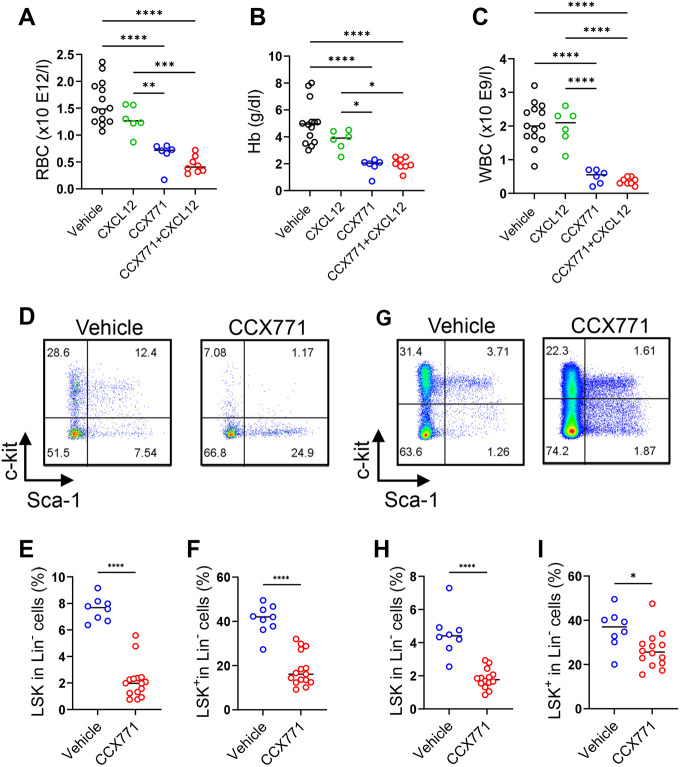
**ACKR3 blockade impairs HSC development in embryos.** (A-C) Haematology blood tests of neonates born to mice injected with vehicle, CXCL12, CCX771 or CCX771 and CXCL12. Red blood cells (RBC; A), haemoglobin (Hb; B) and white blood cells (WBC; C). Vehicle group, *n*=14; CXCL12 group, *n*=6; CCX771 group, *n*=6; CCX771+CXCL12 group, *n*=8. (D-I) Flow cytometry analysis of bone marrow (BM) and fetal liver (FL) of E18.5 embryos from vehicle- or CCX771-injected pregnant mice. Representative flow cytometry plots of Lin^−^ cells in BM (D) showing the percentage of Lin^−^, Sca1^+^ and c-kit^+^ (LSK) cells (E) and Lin^−^, Sca1^−^ and c-kit^+^ (LSK^+^) cells (F) in BM. Representative flow cytometry plots of Lin^−^ cells in FL (G) showing the percentage of LSK cells (H) and LSK^+^ cells (I) in FL. Vehicle group, *n*=8; CCX771 group, *n*=15. Data were pooled from two independent experiments. Graphs show mean. ****P<0.001, ***P<0.005, **P<0.01, *P<0.05 [one-way ANOVA with Tukey's post-test (A-C) or two-tailed unpaired Student's *t*-test (E,F,H,I)]. See also Fig. S3.

As mentioned above, neonates born to mothers injected with CCX771, or CCX771 and CXCL12, were observed to be paler than control neonates. Haematology analysis of neonate blood revealed a substantial reduction in red blood cell numbers in neonates born to mothers injected with CCX771 and CXCL12 (Fig. 4A). Notably, and in contrast to the data relating to weight and length described above, treatment of mothers with ACKR3 blocker alone led to similar levels of anaemia compared with those seen with a combination of blocker and CXCL12 (Fig. 4B). In addition, a general reduction in white blood cell numbers was also seen and, again, this was seen for neonates from both blocker- and blocker-plus-CXCL12-treated mothers (Fig. 4C). Thus, the pale appearance of embryos from mothers injected with CCX771, and CCX771 plus CXCL12, is associated with anaemia.

To determine the basis for this phenotype we next examined BM and fetal liver (FL) HSC numbers in embryos. This analysis focused on vehicle and CCX771-treated mothers, as the haematopoietic anomalies described above were evident in mice treated only with CCX771. In BM, there was a highly significant reduction in the number of Lin^−^Sca1^+^c-Kit^+^ (LSK) HSCs in the embryos from CCX771-treated, compared with vehicle-treated, mothers (Fig. 4D,E). This was also associated with a decrease in the number of Lin^−^Sca1^−^c-Kit^+^ (LS^−^K^+^) HPC within the BM (Fig. 4F). Intriguingly, HSC and HPC populations were also reduced within the FL in embryos from CCX771-treated mothers (Fig. 4G-I) a phenotype that was also apparent in Cyp19-Cre^+/−^ACKR3^fl/fl^ embryos (Fig. S6).

**Fig. 5.**
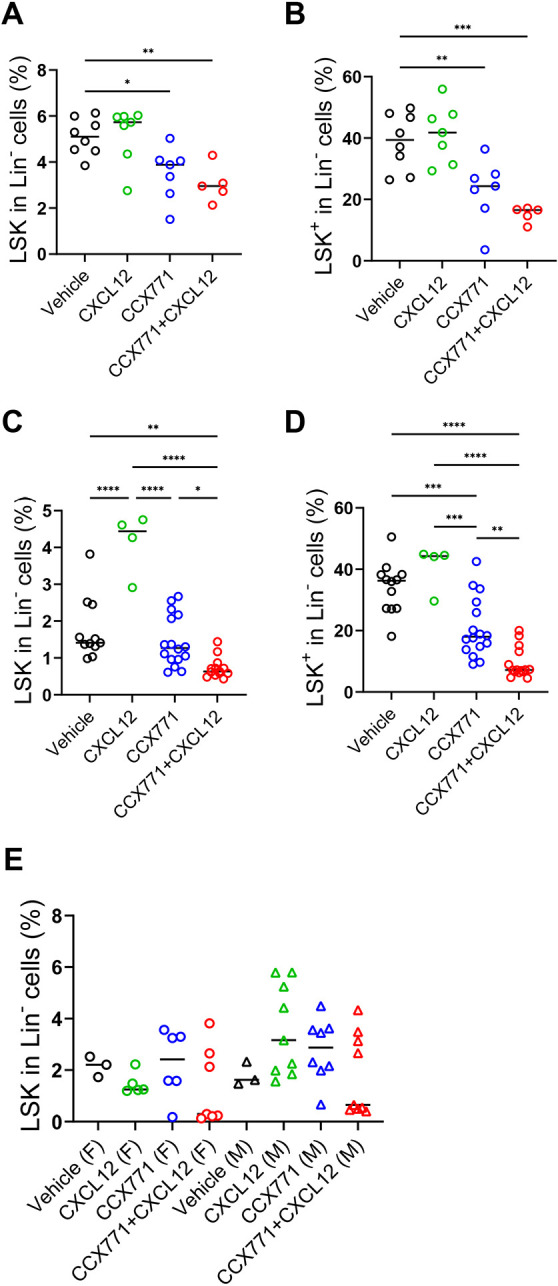
**Maternal ACKR3 blockade is associated with sustained disrupted haematopoiesis after birth.** (A-E) Wild-type pregnant mice were injected with vehicle, CXCL12, CCX771 or CCX771 and CXCL12 (Fig. 3A). (A,B) Analysis of haematopoietic stem cells in neonates. The percentage of Lin^−^, Sca1^+^ and c-kit^+^ cells (LSK) (A) and Lin^−^, Sca1^−^ and c-kit^+^ cells (LSK^+^) (B) in bone marrow (BM) in neonates. Vehicle group, *n*=8; CXCL12 group, *n*=5; CCX771 group, *n*=7; CCX771+CXCL12 group, *n*=7. (C,D) The percentage of BM LSK cells (C) and LSK^+^ cells (D) in Lin^−^ cells in 2-week-old pups. Vehicle group, *n*=11; CXCL12 group, *n*=4; CCX771 group, *n*=16; CCX771+CXCL12 group, *n*=13. (E) The percentage of BM LSK cells in Lin^−^ cells in 7-week-old female (F) or male (M) mice. Vehicle group, *n*=6; CXCL12 group, *n*=14; CCX771 group, *n*=14; CCX771+CXCL12 group, *n*=16. Data were pooled from two independent experiments. Graphs show mean. ****P<0.001, ***P<0.005, **P<0.01, *P<0.05 (one-way ANOVA with Tukey's post-test).

Next, we examined BM haematopoietic parameters in mice at various time points post birth. Neonates born to both CCX771 and CCX771-plus-CXCL12-injected mothers displayed significant reduction in both HSC and HPC numbers (Fig. 5A,B). At 2 weeks, pups born to CCX771-injected mothers had recovered numbers of HSC but remained severely depleted in HPC (Fig. 5C,D). In contrast, both HSC and HPC numbers remained depleted in pups born to mothers treated with a combination of CCX771 and CXCL12. By 7 weeks, HSC and HPC numbers had normalised across progeny from all the treated groups (Fig. 5E).

These data demonstrate that trophoblast ACKR3 blockade is associated with severely compromised haematopoietic development in embryos.

### ACKR3 blockade also has a significant impact on B cell numbers

**Fig. 6.**
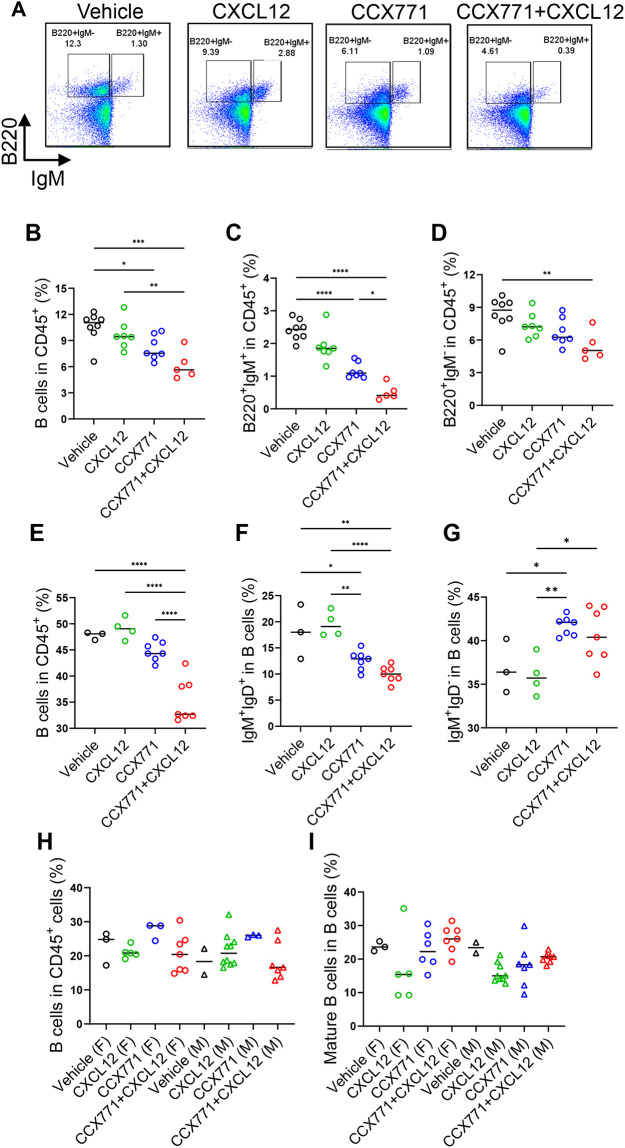
**Maternal ACKR3 blockade is associated with decreased mature B cell numbers in offspring.** (A-D) B cell populations in bone marrow (BM) in neonates born to wild-type pregnant mice injected with vehicle, CXCL12, CCX771 or CCX771 and CXCL12, showing representative flow cytometry plots (A) and the percentage of total B cells (B), immature/mature (B220^+^IgM^+^) cells (C) and Pro/Pre-B cells (B220^+^gM^−^) cells (D). Vehicle group, *n*=8; CXCL12 group, *n*=7; CCX771 group, *n*=7; CCX771+CXCL12 group, *n*=5. (E-G) The percentage of total B cells (E), mature B cells (IgM^+^IgD^+^) cells (F) and immature B cells (IgM^+^IgD^−^) cells (G) in 2-week-old mice. Vehicle group, *n*=3; CXCL12 group, *n*=4; CCX771 group, *n*=7; CCX771+CXCL12 group, *n*=7. (H,I) The percentage of total B cells (H) and mature cells (I) in 7-week-old female (F) or male (M) offspring. Vehicle group, *n*=6; CXCL12 group, *n*=10; CCX771 group, *n*=6; CCX771+CXCL12 group, *n*=14. Data in A-G are representative of two independent experiments. Data in H and I were pooled from two independent experiments. Graphs show mean. ****P<0.001, ***P<0.005, **P<0.01, *P<0.05 (one-way ANOVA with Tukey's post-test). See also Fig. S7.

Given known roles for CXCL12 in B cell biology (D'Apuzzo et al., 1997; Nagasawa et al., 1996), we analysed B cell numbers in offspring. In neonates, there was a general reduction in total B cell numbers, as well as numbers of BM IgM^+^ immature B cells in pups born from CCX771 and CCX771-plus-CXCL12-treated mothers (Fig. 6A-C). Reduction in numbers of B220^+^IgM^−^ B cells (Pro/Pre-B cells) in BM was seen only for pups born to mothers injected with a combination of CCX771 and CXCL12 (Fig. 6D). At 2 weeks, this reduction was seen for total B cell numbers and for mature, but not immature, B cells, which had rebounded in number (Fig. 6E-G; Fig. S7). Both maternal CCX771 and CCX771 plus CXCL12 administration led to significant reduction in mature B cells in 2-week-old offspring (Fig. 6F). By 7 weeks, no differences in B-cell numbers were detected in any of the progeny from any of the treated groups (Fig. 6H,I). These results suggest that trophoblast ACKR3 blockade also has an impact on early B cell development within offspring.

### ACKR3 blockade leads to deficiencies in the inflammatory response in neonates

**Fig. 7.**
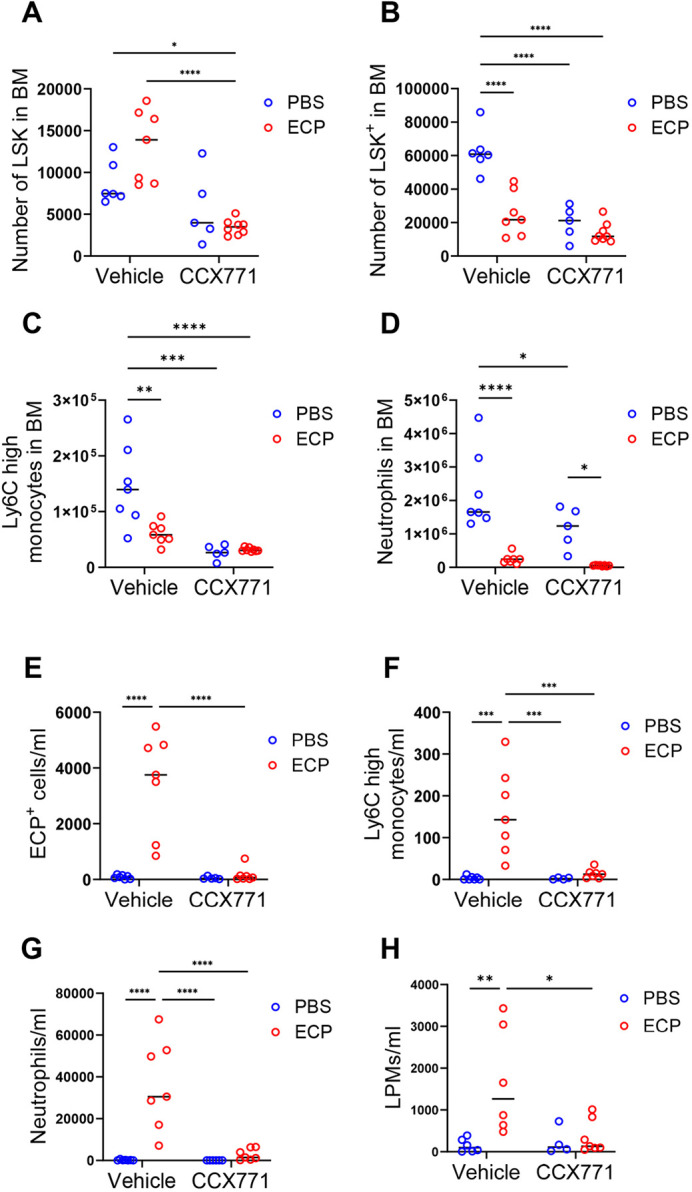
**Maternal ACKR3 blockade is associated with impaired inflammatory responses in offspring.** (A-H) Two-week-old pups born to wild-type pregnant mice injected with vehicle or CCX771 were intraperitoneally injected with PBS or pHrodo-Red-dye-conjugated *E. coli* particles (ECP). The peritoneal lavage and the bone marrow (BM) cells were analysed by flow cytometry 24 h after the injection. Total numbers of Lin^−^, Sca1^+^ and c-kit^+^ (LSK) cells (A) and Lin^−^, Sca1^−^ and c-kit^+^ (LSK^+^) cells (B), Ly6C^high^ monocytes (C) and neutrophils (D) in BM, and ECP^+^ cells (E), Ly6C^high^ monocytes (F), neutrophils (G) and large peritoneal macrophages (LPMs) (H) in peritoneal lavage. Vehicle group, *n*=7 (PBS or ECP); CCX771 group, *n*=5 (PBS) or 8 (ECP). Data are representative of two independent experiments. Graphs show mean. ****P<0.001, ***P<0.005, **P<0.01, *P<0.05 (two-tailed unpaired Student's *t*-test). See also Fig. S8.

Although the suppression of haematopoiesis seen in embryos and neonates from mothers treated with CCX771 resolved by 7 weeks, this leaves a postnatal period in which mice born to mothers treated with CCX771 have significantly reduced numbers of cells from across the haematopoietic spectrum. To examine the implications of this in terms of the ability of preweaned pups to mount protective inflammatory responses we challenged 2-week-old mice, born from mothers injected either with vehicle or CCX771, by intra-peritoneal administration of pHrodo Red-conjugated *Escherichia coli* particles. Pups born to vehicle-injected mothers displayed increased numbers of BM HSC and reduced HPC, with evidence that these cells are mobilised from the BM in response to *E. coli* particle administration. These cell populations in pups born to mothers injected with CCX771 were substantially reduced in number, although not altered, by *E. coli* particles (Fig. 7A,B). The number of Ly6C^high^ monocytes and neutrophils in the BM in pups born to CCX771-treated mothers was significantly lower than those in control pups. Mobilisation of Ly6C^high^ monocytes by *E. coli* particle administration was detected in control pups, but not in pups born to CCX771-treated mothers, whereas mobilisation of neutrophils was observed in both groups. (Fig. 7C,D).

Peritoneal contents were collected by lavage, and *E. coli* particle uptake and recruited cells assessed by flow cytometry (Fig. S8A-E). Although there was substantial uptake of *E. coli* particles by recruited cells in the peritoneum of pups born to control mothers, this was severely depleted in the peritoneum of pups born to CCX771-treated mothers (Fig. 7E). When recruited cells were analysed it was seen that pups born to control-treated mothers demonstrated significant recruitment of Ly6C^high^ monocytes and neutrophils, but that there was minimal recruitment of these cells in pups born to mothers treated with CCX771 (Fig. 7F,G). These results suggest a fundamental inability to mount inflammatory responses in these offspring. Tissue-resident large peritoneal macrophages (LPMs) were increased in control pups by the administration of the *E. coli* particles; however, LPMs in pups born to CCX771-treated mothers were not altered (Fig. 7H). In keeping with the data described above, there was a reduction in peritoneal B cell numbers in pups born to CCX771-treated mothers, but these cells appear to be unaffected by the presence or absence of *E. coli* particles (Fig. S8F).

Overall, these data indicate that the depletion of haematopoietic stem and progenitor cells seen in neonates from CCX771-treated mothers is associated with a profound inability to recruit inflammatory cells in response to peritoneal bacterial challenge.

## DISCUSSION

Here, using a well-characterised pharmacological blocker and confirmatory genetic experiments, we demonstrate that trophoblast ACKR3 has an important role in restricting the movement of CXCL12 across the trophoblast barrier into the embryonic circulation. It is clear from our analyses that the effects of interfering with trophoblast ACKR3 are profound in terms of altering developmental parameters, with pups born to ACKR3-blocker-treated mothers being characterised by growth retardation and marked alterations to haematopoietic system development. These phenotypes continue through the neonatal period and, although haematopoietic parameters are normalised by 7 weeks, the reduced weight seen in mice born to ACKR3-blocker-treated mothers is not fully recovered over time. The normalisation of haematopoietic parameters is fully in keeping with the self-renewal potential of the haematopoietic system.

Notably, administration of CCX771 alone to mothers resulted in a significant reduction of HSC and HPC numbers and associated severe anaemia, whereas administration of a combination of CCX771 and CXCL12 to mothers was required for significant effects on more general developmental parameters including weight and length. This suggests that HSC migration within the embryo is more susceptible to low level maternal CXCL12 entry into the embryo than other developmental processes.

A previous publication (Sierro et al., 2007) on ACKR3-deficient mice reported no alterations in overall B cell numbers in the full knockout mice. In our study, overall B cell numbers were marginally reduced in mice treated only with CCX771 but substantially reduced in mice treated with a combination of CCX771 and maternally-administered CXCL12. In addition, in our study, the impact of CCX771 was specifically seen for a subpopulation of B cells characterised by IgM positivity, and no significant difference in the numbers of IgM^−^ B cells was observed in CCX771-treated mice. These differences in experimental approach, and analysis, are likely to explain the differences between our two studies. Furthermore, Sierro and colleagues reported no difference in neutrophils in ACKR3-deficient mice, although this was not quantified in the paper and thus its implications for the current study are not clear.

Crucially, our data indicate that there is a significant postnatal period during which mice born to mothers with impaired ACKR3 activity have significant deficiencies within their haematopoietic system. Importantly this is reflected in a marked inability of these mice to mount protective inflammatory responses to peritoneal bacterial challenge. This suggests that altered trophoblast ACKR3 activity would leave offspring susceptible to potentially lethal postnatal infections of the peritoneal cavity and other tissues.

It is worth highlighting that this study could not be done with fully ACKR3-deficient mothers, as homozygous ACKR3 deficiency is perinatally lethal. Thus, the availability of a high-quality ACKR3 antagonist, which does not significantly cross the placenta, has afforded us a unique opportunity to analyse chemokine compartmentalisation in the context of ACKR3. Crucially, where CCX771 is observed to cross the placenta, the levels do not correlate with the phenotypes observed, indicating that inhibition of ACKR3 within the embryo-proper is not contributing to the phenotypes observed.

Interestingly, although we have demonstrated a similar role for ACKR2 in the placenta, it is important to note that the localization of ACKR2 and ACKR3 in the placenta is different. ACKR3 is expressed in trophoblasts in the labyrinth, whereas ACKR2 expression is observed predominantly on trophoblasts in the junctional zone (Lee et al., 2019). Previous studies have demonstrated that CXCL12 is expressed in trophoblasts (Ren et al., 2012; Wu et al., 2004) rather than the maternal decidua, whereas CCL2, which is one of the ligands for ACKR2, is expressed in maternal decidua (Lin et al., 2022) and also seen at high levels in the blood of inflamed or infected mothers. This suggests that ACKR2 mainly scavenges its ligand arising in the placenta from maternal blood or the maternal decidua. In contrast, ACKR3 is required to prevent CXCL12 derived from intra-placental sources including trophoblasts, as well as from maternal blood, from infiltrating into the embryonic circulation. Thus, although operating in a mechanistically similar manner, ACKR2 and ACKR3 display discrete domains of expression, and therefore function, within the placenta, which reflect the expression patterns of the ligands.

Overall, therefore, our results indicate a crucial role for trophoblast ACKR3 in protecting the embryo from maternal CXCL12, and that interference with ACKR3 function leads to severe developmental/haematopoietic consequences for offspring. These results suggest that defects in trophoblast ACKR3 in humans, associated with impaired expression levels or function, may lead to developmental/haematopoietic abnormalities similar to those observed in the present study. Notably, previous studies have shown that decreased ACKR3 expression in placentas and increased CXCL12 levels in blood were observed in pregnant women diagnosed with preeclampsia (Lu et al., 2016; Schanz et al., 2011). Also, it is likely that this will be exacerbated by a range of maternal inflammatory and immune diseases, many of which are known to be associated with enhanced levels of plasma CXCL12 (Cruz-Orengo et al., 2011; Hansen et al., 2006; He et al., 2016; Ikawa et al., 2021). Further studies are required to understand the role of ACKR3 on pregnancy/fetus development in humans. However, our findings provide new insights into the regulation of chemokines at the maternal-fetal interface and the importance of this regulation for development of the haematopoietic and immune systems in pups.

## MATERIALS AND METHODS

### Mice

WT C57BL/6J mice were from Charles River Laboratories. ACKR3-GFP reporter mice were from The Jackson Laboratory (C57BL/6-Ackr3tm1Litt/J) (Cruz-Orengo et al., 2011). Cyp19Cre mice were a generous gift from Prof. Gustavo Leone (Wenzel and Leone, 2007) and ‘floxed’ ACKR3 mice, B6.Cg-*Ackr3*^tm1Fma^/J, were obtained from The Jackson Laboratory. We used 7-10 week-old female mice for timed mating. Mice were maintained in specific pathogen-free facilities at the Beatson Institute and University of Glasgow. All experiments were performed under a UK Home Office Project License and approved by local ethical review committee at the University of Glasgow.

### *In vivo* experimental procedures

Ackr3^GFP/+^ females mated with genotype-matched males were injected with 0.5 μg of AlexaFluor 647-conjugated human CXCL12, and 1 and 3 h after the injection placentas and embryonic blood were obtained for immunofluorescence staining and ELISA, respectively. For *in vivo* blockade of ACKR3, timed pregnant WT females were subcutaneously injected with either Captisol (vehicle) or 30 mg/kg of CCX771 (Zabel et al., 2009) between E12.5 and E18.5. In addition, PBS or 0.5 μg of recombinant mouse CXCL12 were intravenously injected into pregnant mice between E13.5 and E18.5. Pregnant females were weighed and monitored daily for parturition. For induction of an inflammatory response, 2-week-old offspring were intraperitoneally injected with PBS or 100 μg of Deep Red *E. coli* Bioparticles. Then 24 h after the injection peritoneal lavage was carried out and BM cells were obtained and analysed by flow cytometry. Pups were monitored daily and weighed weekly before the injection or tissue collection. Length of pups was defined as the distance from the tip of the nose to the start of tail. See Table S1 for reagents and resources.

### Digestion of placentas

For flow cytometry, placentas were separated into maternal decidua and placenta proper with fine forceps using a dissecting microscope. Tissues were incubated in 1 ml of HBSS-based digestion cocktails containing 800 μg/ml of dispase-II, 200 μg/ml of collagenase-P and 100 μg/ml of DNase-I at 37°C at 1000 rpm for 1 h on a temperature-controlled shaker. Then 1 ml of RPMI 1640 with 10% fetal calf serum (FCS) was added to digested tissues to neutralize enzymes and the tissues were filtered through 100 μm cell strainers. After washing cells with PBS, cells were incubated with red blood cell lysis buffer and resuspended in fluorescence-activated cell sorting (FACS) buffer (1% FCS and 2 mM EDTA in PBS).

### Quantitative real-time PCR

Total RNAs were isolated from maternal decidua and placentas using a PureLink RNA Mini Kit according to the manufacturer's instructions. We used 100 ng of RNAs for synthesis of cDNA using a High Capacity cDNA reverse transcription Kit (Dyer et al., 2019). qRT-PCR was performed using a PerfeCTa SYBR Green SuperMix on the ABI 7900HT. Data were normalized to the expression of *Gapdh*. Primer sequences for qRT-PCR: *ACKR3* Forward: 5′-GTGTCCCACCATGCCTAACA-3′, Reverse: 5′-TGTAGCAGTGCGTGTCGTAG-3′; *ACKR3* (standard) Forward: 5′-GTCACTTGGTCGCTCTCCTC-3′, Reverse: 5′-TGGAAGCAGATGTGACCGTC-3′; *Cxcl12* Forward: 5′-AGAGCCAACGTCAAGCATCT-3′, Reverse: 5′-TAATTTCGGGTCAATGCACA-3′; *Cxcl12* (standard) Forward: 5′-TGACGGTAAACCAGTCAGCC-3′, Reverse: 5′-TACCGTCAGGTTTGAGCACC-3′; *Gapdh* Forward: 5′-AATGTGTCCGTCGTGGATCT-3′, Reverse: 5′-AGACAACCTGGTCCTCAGTG-3′; *Gapdh* (standard) Forward: 5′-ACATCATCCCTGCATCCACT-3′, Reverse: 5′-GAGTTGGGATAGGGCCTCTC-3′.

### Flow cytometry

Cell suspensions were incubated with Fc-block for 10 min at 4°C, and then stained with antibodies in FACS buffer for 30 min at 4°C. To define trophoblast cells, digested placental cells were stained with PerCP-Cy5.5-anti-mouse CD45, APC-anti-mouse CD9 and PE-anti-mouse CD71. To define HSC, BM and FL cells were stained with APC-CD45, BV421-CD117 (c-Kit), PE-Cy7-Ly-6A/E (Sca1) and lineage markers including biotin-anti-mouse CD3ε, B220, CD19, NK1.1, F4/80, CD11c and Ly-6G/Ly-6C (Gr-1). PerCP-Cy5.5-conjugated streptavidin was used for detection of biotinylated antibodies. To define monocytes, macrophages and neutrophils in peritoneal lavage and BM, cells were stained with anti-mouse BV785-CD11b, APC-Fire750-Ly6c, BV650-CD19, PE-Cy7-F4/80, BUV395-CD45 and BUV805-Ly6G. To define B cells, cells were stained with anti-mouse BV605-CD45, PerCP-Cy5.5-CD19, FITC-B220, BV421-IgD and APC-IgM. After staining with antibodies, cells were incubated with fixable viability dye eFluor780 or eFluor506 for 20 min at 4°C. Cells obtained were washed and then fixed with 2% paraformaldehyde (PFA). Flow data were obtained using a LSRII (BD Bioscience) or Fortessa flow cytometer (BD Bioscience) and analysed with FlowJo software Ver.10. See Table S1 for antibodies.

### Immunofluorescence staining

Placentas were fixed with 4% PFA at 4°C overnight. The tissues were incubated with 30% sucrose for 2 h at 4°C and then frozen in OCT. Frozen tissues were sectioned to 7 μm using a cryostat (Model OTF, Bright Instruments). Sections were incubated with blocking buffer (1% bovine serum albumin and 0.5% Tween-20 in PBS) for 15 min at room temperature (RT), and then stained with biotin-anti-mouse CD9 or rabbit anti-MCT4 at 4°C overnight. Sections were washed and incubated with Streptavidin-Alexafluor 594 or Alexafluor 594 anti-rabbit IgG. See Table S1 for antibodies. Images were obtained at 40× magnification using the Axioimager M2 (ZEISS).

### Human samples

Placental samples were obtained at elective caesarean sections and placed in 10% neutral buffered formalin for fixation, processing and embedding. Written informed consent was obtained and the project was approved by the West of Scotland Research Ethics Committee (reference 17/WS/0174 and 19/WS/0111).

For immunohistochemistry, sections were deparaffinised as above, then incubated with Bloxall to block endogenous peroxidase and heated in retrieval buffer (10 mM citric acid buffer, pH 6.0). The sections were incubated with 2.5% horse serum for 30 min at RT followed by staining with polyclonal anti-human ACKR3 antibody or isotype control at 4°C overnight. They were then incubated with horseradish peroxidase-conjugated secondary antibody (Table S1) for 30 min at RT and visualized using 3,3-diaminobenzibine for 30 s at RT. Sections were counterstained with haematoxylin followed by dehydration using ethanol and xylene, and 40× magnification stitched images were obtained using an EVOS FL auto2 microscope (Invitrogen).

### Haematoxylin and Eosin (H&E) staining

Placentas were fixed with 4% PFA at 4°C overnight. For H&E staining, the tissues were processed, embedded in paraffin and sectioned at 5 μm using a microtome (Finesse 325, Thermo Fisher Scientific). The sections were deparaffinised in xylene and rehydrated in serial ethanol gradients and then stained with H&E, and then 20× magnification ‘stitched images’ were obtained using an EVOS FL auto2 microscope.

### ELISA

Embryonic blood was collected, following decapitation, into 1.5 ml Eppendorf tubes filled with 20 μl of 0.2 M EDTA. The blood was centrifuged at 1500 ***g*** for 10 min at 4°C to collect the plasma. The concentrations of CXCL12 were measured using mouse or human CXCL12 Duoset ELISA kits according to the manufacturer's instructions. Total protein concentrations were measured using a Pierce BCA Protein Assay kit. The concentration of CXCL12 was normalized to the overall concentration of total proteins.

### Haematology study

Pups were euthanized with Dolethal at postnatal day 1 or 2. Blood was collected from jugular veins into EDTA-coated tubes filled with 20 μl of 0.2 M EDTA. Haematological analysis was performed by University of Glasgow Veterinary diagnostic services.

### Statistics

Two-tailed unpaired Student's *t*-test, one-way ANOVA followed by Tukey's test (multiple comparisons) or two-way ANOVA followed by Bonferroni's test (multiple comparisons) were performed using Prism9. *P*-values of less than 0.05 were considered statistically significant. All data are presented as mean±s.d.

## Supplementary Material



10.1242/develop.202333_sup1 Supplementary information
