# Supplementary material for: Disruption of placental ACKR3 impairs growth and hematopoietic development of offspring
Source: Development. 2024 Feb 23;151(4):dev202333. doi: 10.1242/dev.202333 (PMC10911115; doi:10.1242/dev.202333)
Supplement: Supplementary information [file develop-151-202333-s1.pdf]

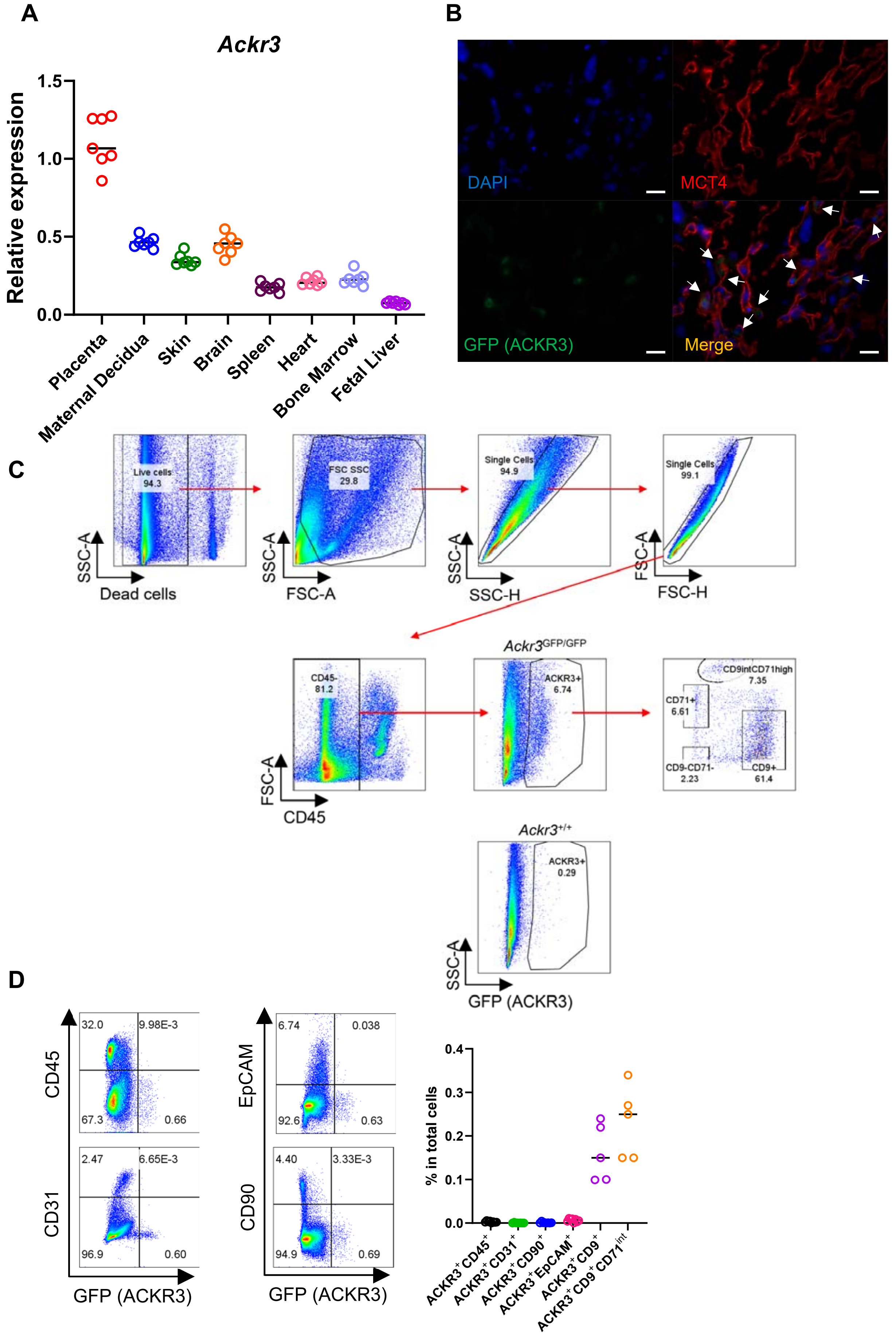

Fig. S1. ACKR3 expression on trophoblasts. (Related to Figure 1B and C)

(A) ACKR3 expression by QPCR in tissues from wild-type E18.5 embryos. n=7.

(B) Representative images of labyrinth in placentas of ACKR3 reporter mice at E15.5. Sections were stained with a syncytiotrophoblast marker, MCT4. Scale bars indicate 20 µm. n=5.

(C) Gating strategy to determine the expression of ACKR3 on trophoblast cells in the placenta. n=5

(D) Representative flow cytometry plots of ACKR3 (GFP)-positive cells in the placenta. The graph shows percentages of ACKR3^{+} and CD45^{+}, CD31^{+}, CD90^{+}, EpCAM^{+}, CD9^{+} or CD71^{+} cells in fetal side of placentas. n=5.

A

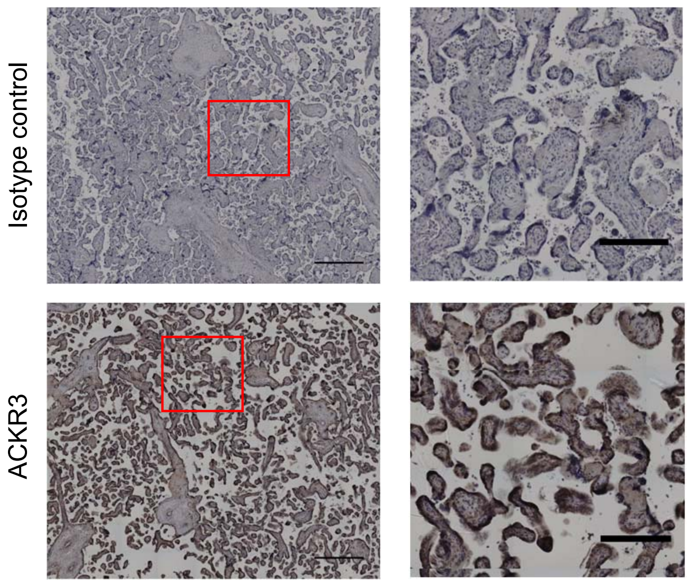

B

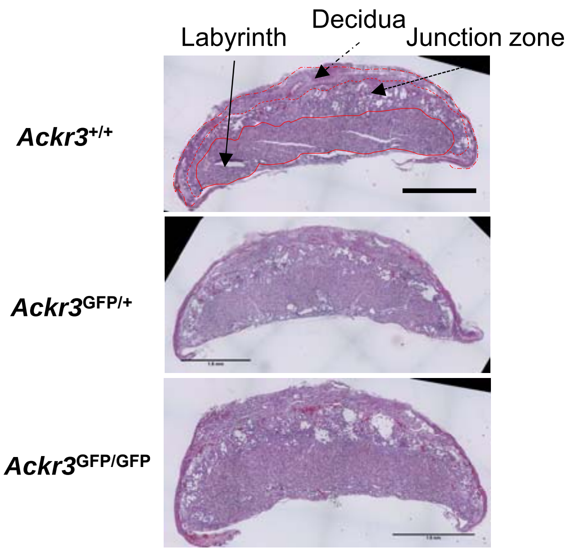

Fig. S2. Human trophoblast cells express ACKR3. (Related to Figure 1D and F)

(A) Human placentas were stained with anti-human ACKR3 antibody. Hematoxylin was used for counterstaining. Scale bars indicate 0.5 cm (Left) and 0.25 cm (Right). n=3.

(B) Representative images of HE staining of placentas of *Ackr3*$^{+/+}$, *Ackr3*$^{GFP/+}$ and *Ackr3*$^{GFP/GFP}$ embryos. The proportion of decidua, junction zone and labyrinth were measured using ImageJ. Scale bars indicate 1.5 mm.

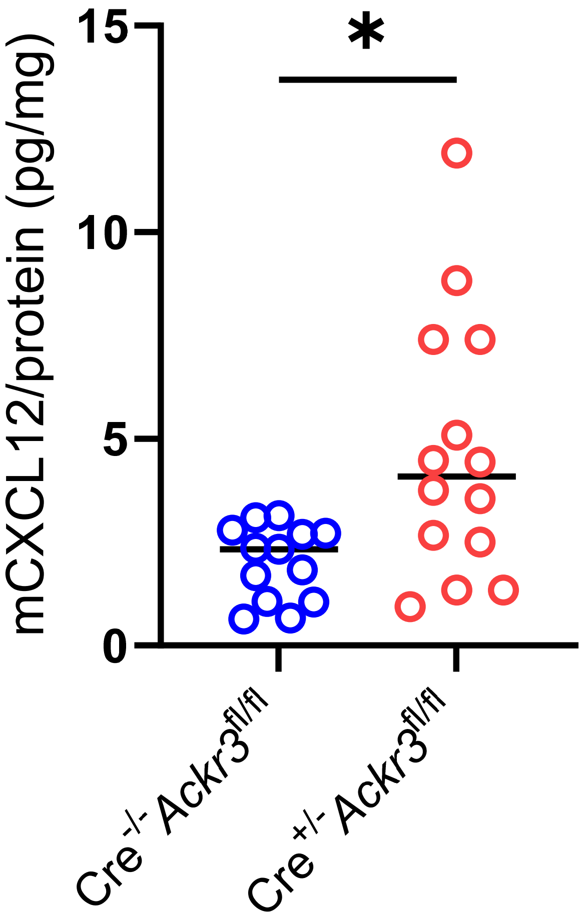

Fig S3. CXCL12 levels in the plasma of embryos with either Cre$^{-/-}$Ackr3$^{fl/fl}$ or Cre$^{+/-}$Ackr3$^{fl/fl}$ genotypes. Data are presented as pg/mg (total protein). *p<0.05 (unpaired, two-tailed Student's t-test).

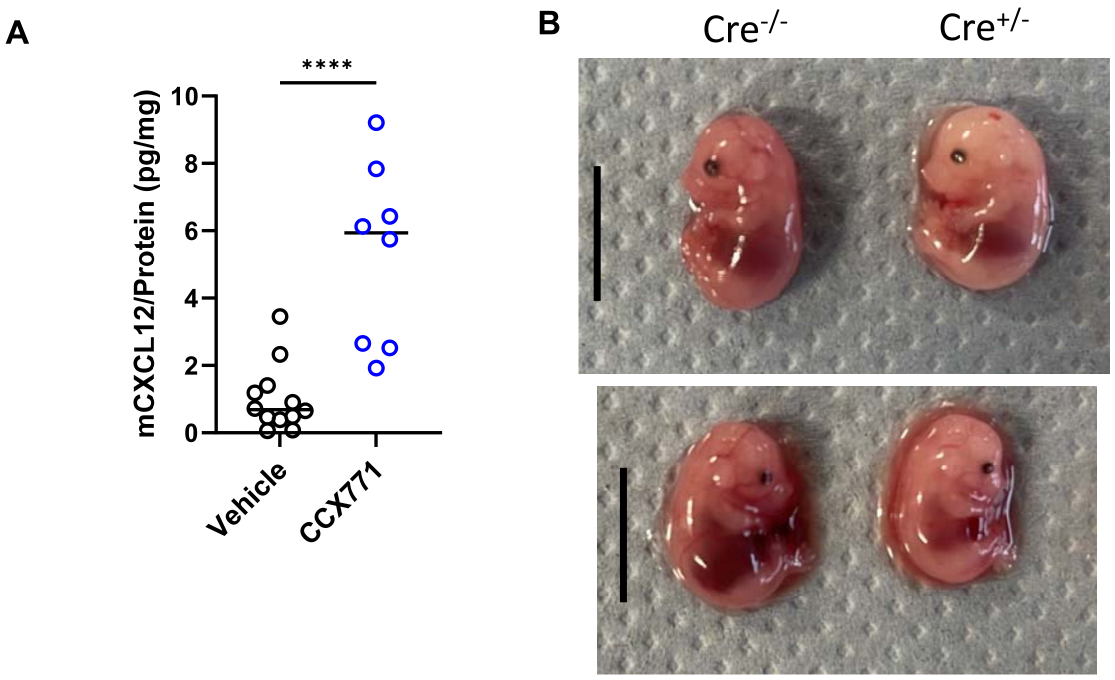

Fig S4.

(A) CXCL12 levels in embryonic blood at E18.5 after the administration of CCX771 to mother mice. Vehicle group, n=12, CCX771 group, n=8. ****$P<0.0001$ (unpaired, two-tailed Student's t-test). (B) Pictures of E15.5 embryos in $Cre^{+/-}$ $Ackr3^{fl/fl}$ females mated with $Cre^{-/-}$ $Ackr3^{fl/fl}$ males. Scale bars indicate 1 cm.

A

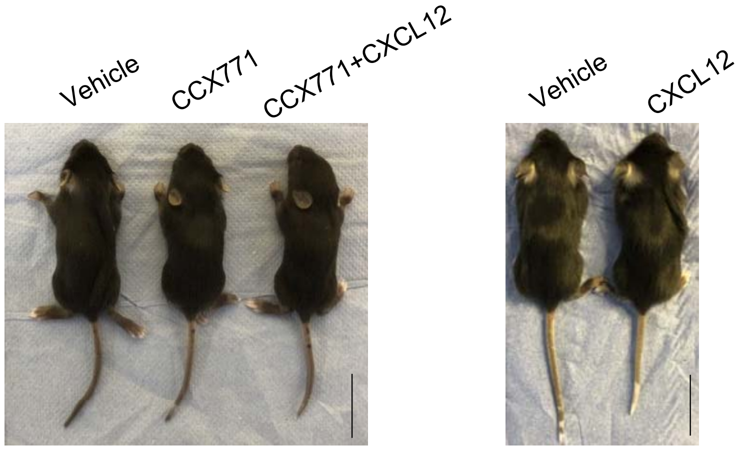

B

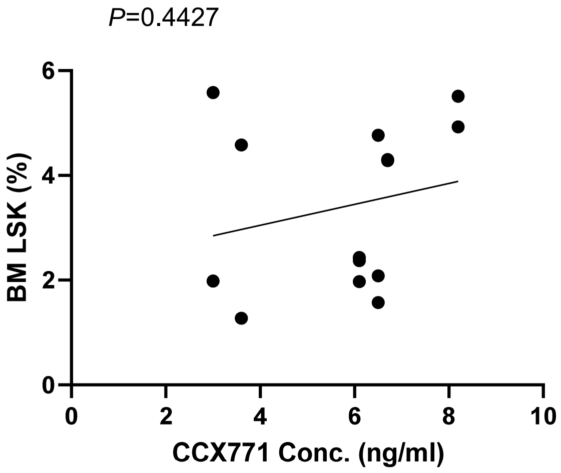

Fig. S5. Maternal injection of CCX771 does not infiltrate into embryonic circulation. (Related to Figure 3 and 4)

(A) Representative images of 2-week-old offspring born to Vehicle, CXCL12, CCX771 or CCX771 and CXCL12 injected pregnant mice. Scale bars indicate 2 cm.

(B) CCX771 concentrations in plasma in E18.5 embryos are plotted against the percentage of LSK cells in bone marrow with the best fit lines and *P* values calculated using simple linear regression. The percentage of LSK cells versus CCX771 concentration (R2=0.049, slop=0.1995, y-intercept=2.25).

Development • Supplementary information

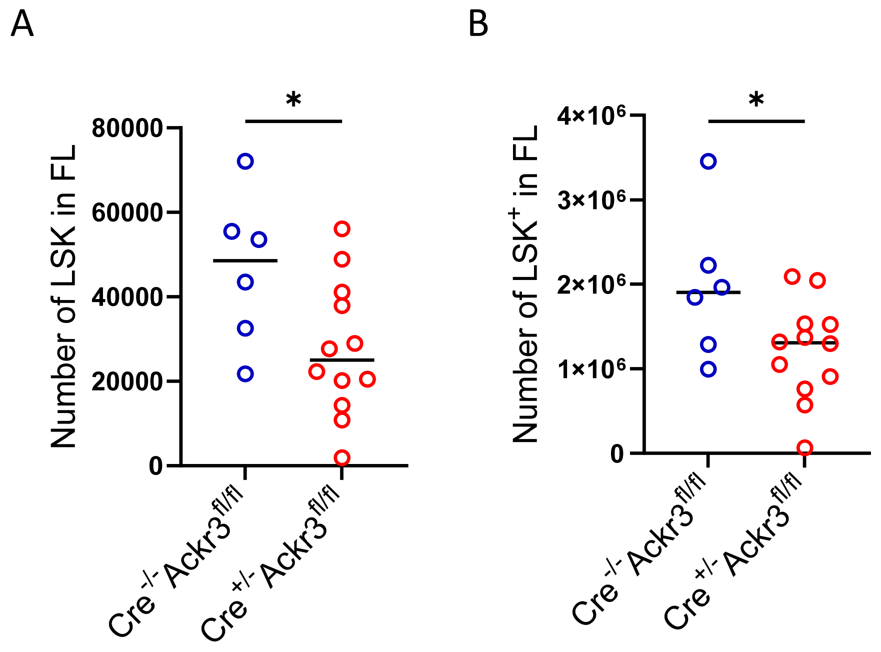

Fig. S6. Hematopoietic stem (A) and progenitor (B) cell numbers in the fetal livers of E15.5 embryos of either $Cre^{-/-}Ackr3^{fl/fl}$ or $Cre^{+/-}Ackr3^{fl/fl}$ genotypes. $Cre^{-/-}$ $Ackr3^{fl/fl}$ group, n=6, $Cre^{+/-}$ $Ackr3^{fl/fl}$ group, n=12. *$p<0.05$ (unpaired, two-tailed Student's t-test).

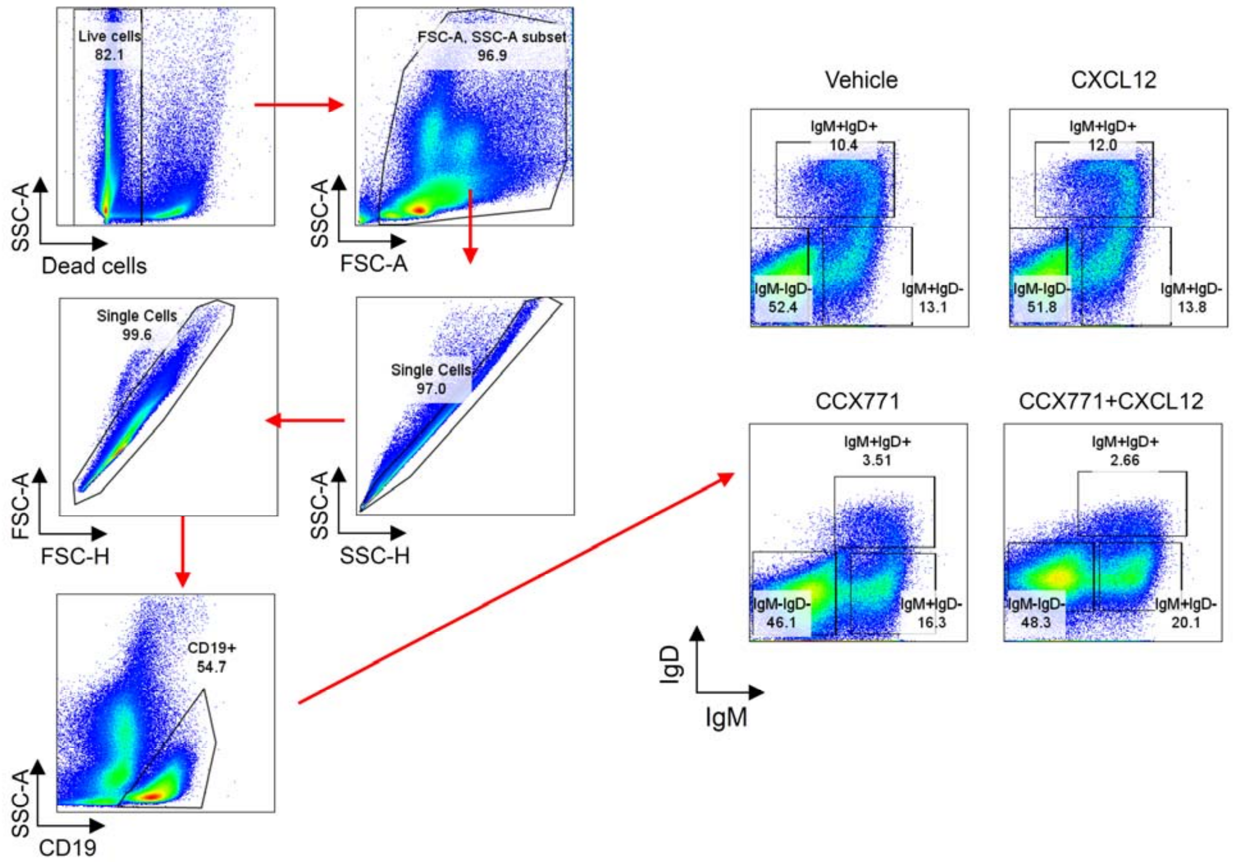

Fig. S7. Gating strategy of B cells in young mice. (Related to Figure 6)
Gating strategy to define bone marrow B cells in 2-week-old pups. B cells were defined as $CD45^+CD19^+$ cells.

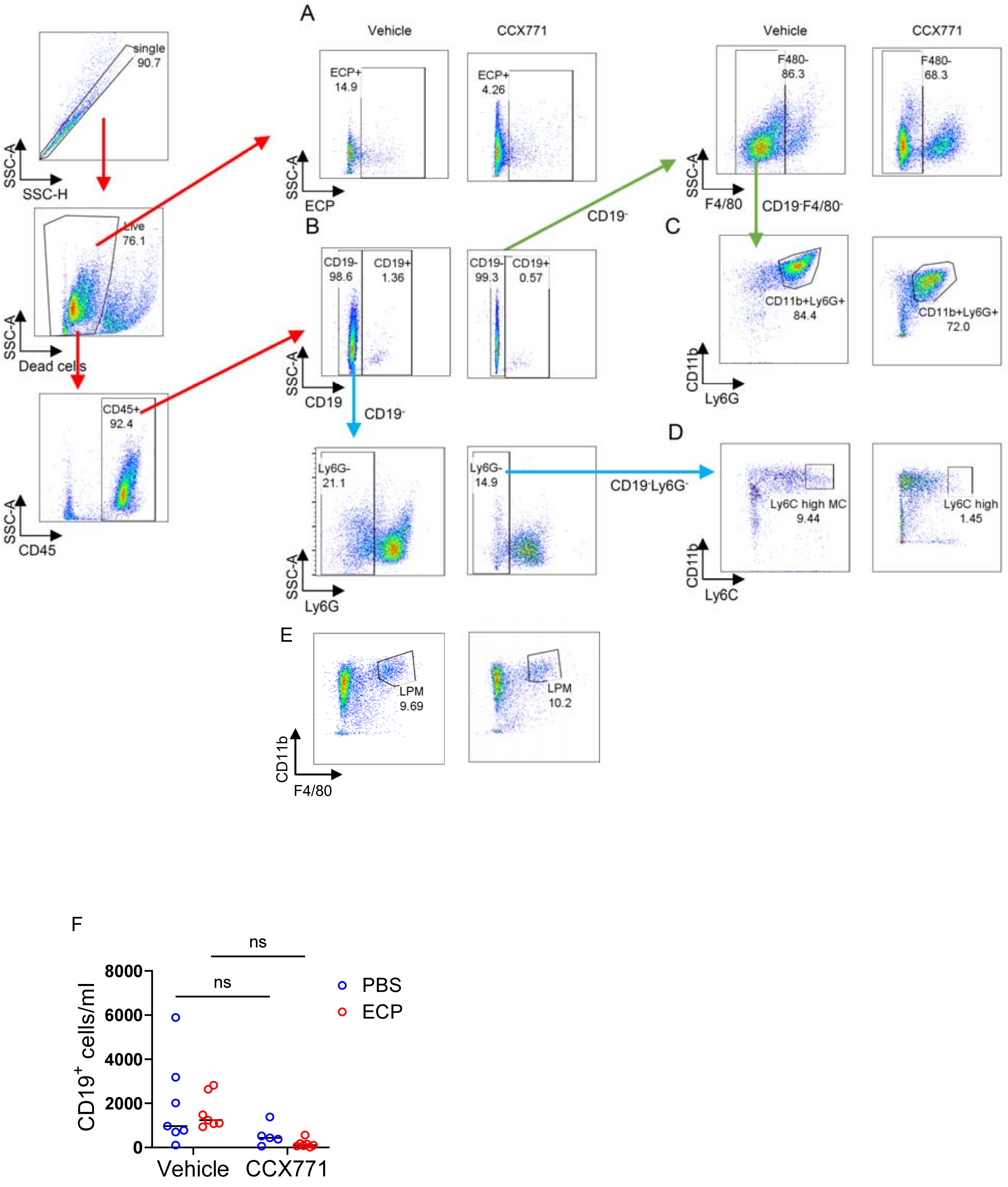

Fig. S8. Gating strategy for peritoneal cells. (Related to Figure 7)

2-week-old pups born to vehicle, or CCX771, injected mothers were intraperitoneally injected with PBS (50 µl) or Deep Red *E.coli* bioparticles (ECP) (100 µg/50 µl). (A-E) The gating strategy to define ECP^{+} cells (A), CD19^{+} cells (CD45^{+}CD19^{+}) (B), Ly6C high monocytes (CD45^{+}CD19^{-}CD11b^{+}Ly6C^{high}) (C), neutrophils (CD45^{+}CD19^{-} F4/80 $^{-}$CD11b^{+}Ly6G^{+}) (D) and Large peritoneal macrophages (LPMs) (E) in the peritoneal lavage. (F) Number of B cells (CD19^{+} cells) in the peritoneal lavage. ns; not significant.

Table S1. Information of antibodies, reagents and kits used for this study.

Antibodies	Company	Catalog number; RRIDs
anti-mouse CD45 (PerCP-Cy5.5); clone 30-F11	Biolegend	Cat # ab1791; RRID: AB_893340, 1:200 dilution
anti-mouse CD9 (APC); clone MZ3	Biolegend	Cat# 124811; RRID: AB_2783070, 1:200 dilution
anti-mouse CD71 (PE); clone RI7217	Biolegend	Cat# 113807; RRID: AB_313568, 1:200 dilution
anti-mouse CD3ε (Biotin); clone 145-2C11	Biolegend	Cat# 100304; RRID: AB_312669, 1:200 dilution
anti-mouse B220 (Biotin); clone RA3-6B2	Biolegend	Cat# 103204; RRID: AB_312989, 1:200 dilution
anti-mouse CD19 (Biotin); clone 6D5	Biolegend	Cat# 115504; RRID: AB_313639, 1:200 dilution
anti-mouse NK1.1 (Biotin); clone PK136	Biolegend	Cat# 108704; RRID: AB_313391, 1:200 dilution
anti-mouse F4/80 (Biotin); clone BM8	Biolegend	Cat# 123106; RRID: AB_893501, 1:200 dilution
anti-mouse CD11c (Biotin); clone N418	Biolegend	Cat# 117304; RRID: AB_313773, 1:200 dilution
anti-mouse Ly-6G/Ly-6C (Gr-1) (Biotin); clone RB6-8C5	Biolegend	Cat# 108404; RRID: AB_313369, 1:200 dilution
anti-mouse CD45 (APC); clone 30-F11	Biolegend	Cat# 147708; RRID: AB_2563540, 1:200 dilution
anti-mouse CD117 (c-Kit) (Brilliant Violet 421); clone 2B8	Biolegend	Cat# 105827; RRID: AB_10898120, 1:200 dilution
anti-mouse Ly-6A/E (Sca-1) (PE-Cy7); clone D7	Biolegend	Cat# 108114; RRID: AB_493596, 1:200 dilution
anti-mouse CD11b (Brilliant Violet 785); clone M1/70	Biolegend	Cat# 101243; RRID: AB_2561373, 1:200 dilution
anti-mouse Ly6C (APC-Fire 750); clone HK1.4	Biolegend	Cat# 128046; RRID: AB_2616731, 1:200 dilution
anti-mouse F4/80 (PE-Cy7); clone BM8	Biolegend	Cat# 123114; RRID: AB_893478, 1:200 dilution
anti-mouse CD45 (Brilliant Violet 605); clone 30-F11	Biolegend	Cat# 103155; RRID: AB_2650656, 1:200 dilution
anti-mouse CD19 (PerCP-Cy5.5); clone 6D5	Biolegend	Cat# 115534; RRID: AB_2072925, 1:200 dilution
anti-mouse B220 (FITC); clone RA3-6B2	Biolegend	Cat# 103206; RRID: AB_312991, 1:200 dilution
anti-mouse IgD (Brilliant Violet 421); clone 11-26c.2a	Biolegend	Cat# 405725; RRID: AB_2562743, 1:200 dilution
anti-mouse IgM (APC); clone RMM-1	Biolegend	Cat# 406509; RRID: AB_315059, 1:200 dilution
anti-mouse CD45 (BUV 395); clone 30-F11	BD Biosciences	Cat# 564279; RRID: AB_2651134, 1:200 dilution
anti-mouse Ly6G (BUV 805); clone 1A8	BD Biosciences	Cat# 741994; RRID: AB_2871294, 1:200 dilution
Rabbit anti-MCT4	Merck	Cat# AB3314P, 1:100 dilution
anti-mouse CD9 (Biotin); clone MZ3	Biolegend	Cat# 124803; RRID: AB_2076036, 1:100 dilution
Goat anti-Rabbit IgG (H+L) Highly Cross-Adsorbed Secondary Antibody, Alexa Fluor™ 594	Invitrogen	Cat# A-11037, 1:1000 dilution
anti-human ACKR3 antibody Lot: A106375	ATLAS ANTIBODIES	Cat# HPA049718, 1:50 dilution
Rabbit IgG, control antibody	Vector	Cat# I-1000-5
**Chemicals, enzymes and other reagents**	**Company**	**Catalog number; RRIDs**
Fixable Viability Dye eFluor780	eBioscience	Cat# 65-0865-18
Fixable Viability Dye eFluor506	eBioscience	Cat# 65-0863-18
pHrodo™ Deep Red E. coli BioParticles™ Conjugate for Phagocytosis	Invitrogen	Cat# P35360
Dispase II	Thermo Fisher SCIENTIFIC	Cat# 17105041
Collagenase P from *Clostridium histolyticum*	Roche	Cat# 11215809103
DNase I Grade II, from bovine pancreas	Roche	Cat# 10104159001
Recombinant human CXCL12	R&D SYSTEMS	Cat# 350-NS-010/CF
Recombinant mouse CXCL12	R&D SYSTEMS	Cat# 460-SD-010/CF
h-SDF-1 alpha (AF647) synthetic	ALMAC	Cat# CAF-11
ProLong Gold Antifade mounting media with DAPI	Invitrogen	Cat# P10144
CCX771	ChemoCentryx	DOI:10.4049/jimmunol.0900269.
PerCP-Cy5.5 Streptavidin	Biolegend	Cat# 405214; RRID: AB_2716577
Alexa Flour 594 Streptavidin	Biolegend	Cat# 405240
**Software**	**Company**	**Catalog number; RRIDs**
FlowJo software Ver. 10	Tree Star	https://www.flowjo.com
GraphPad Prism v9	GraphPad	https://www.graphpad.com
Image J 1.52a	National Institutes of Health	https://imagej.nih.gov/ij/index.html
**Kits**	**Company**	**Catalog number**
PureLink RNA Mini Kit	Invitrogen	Cat# 12183018A
Human CXCL12/SDF-1 DuoSet ELISA	R&D SYSTEMS	Cat# DY350
Mouse CXCL12/SDF-1 DuoSet ELISA	R&D SYSTEMS	Cat# DY460
PerfeCTa® SYBR® Green FastMix	Quanta Biosciences	Cat# 95073-012
High capacity RNA-to-cDNA	Applied Biosystems	Cat# 4387406
ImmPRESS® HRP Horse Anti-Rabbit IgG	Vector	Cat# MP-7401
Polymer Detection Kit	Thermo Scientific	Cat# 23227
ImmPACT® DAB	Vector	Cat# SK-4105
