# Supplementary material for: Disruption of placental ACKR3 impairs growth and hematopoietic development of offspring
Source: Development. 2024 Feb 23;151(4):dev202333. doi: 10.1242/dev.202333 (PMC10911115; doi:10.1242/dev.202333)
Supplement: Supplementary file 1 [file develop-151-202333_review_history.pdf]

Disruption of placental ACKR3 impairs growth and hematopoietic development of offspring

Ayumi Fukuoka, Gillian J Wilson, Elise Pitmon, Lily Koumbas-Foley, Hanna Johnsson, Marieke Pingen and Gerard Graham
DOI: 10.1242/dev.202333

Editor: Liz Robertson

Review timeline

Original submission:	23 June 2023
Editorial decision:	3 July 2023
Rebuttal submitted:	6 July 2023
Editorial response:	19 July 2023
Resubmission:	4 September 2023
Editorial decision:	17 November 2023
First revision received:	14 December 2023
Editorial decision:	22 January 2024
Second revision received:	24 January 2024
Accepted:	25 January 2024

Original submission

First decision letter

MS ID#: DEVELOP/2023/202119

MS TITLE: Disruption of maternal ACKR3 has profound effects on embryos and offspring

AUTHORS: Ayumi Fukuoka, Gillian J Wilson, Elise Pitmon, lily Koumbas Foley, Hanna Johnsson, Marieke Pingen, and Gerry Graham

Many thanks for submitting your manuscript to Development. I have read it carefully and I am sorry to say that I am returning it without review. The guidelines to our reviewers would almost certainly lead to the rejection of the paper after the review process. I hope that this rapid decision will give you the opportunity to submit your work to a more suitable journal without further delay.

Rebuttal Letter

Many thanks for the email and for responding to our submission so promptly. I don't want to be a nuisance but it would be valuable to get some idea about what was missing from the paper that would make it acceptable for review in Development. We understand that the basic mechanism we report has previously been published by us but the molecules are different and the phenotypes we report in the current paper are considerably more stark, with quite profound implications, compared to the phenotype in the original paper.

We had hoped that the impressive nature of the phenotype would be enough to persuade you that the paper should be reviewed. I know that you are very busy but any feedback would be gratefully received and if there is any scope for requesting a reconsideration of this decision then please let me know.

© 2024. Published by The Company of Biologists under the terms of the Creative Commons Attribution License (https://creativecommons.org/licenses/by/4.0/).

Rebuttal Response Letter

Let me first apologise for the delay in getting back to you.

At the time of submission I discussed your manuscript with a member of the Editorial Board and the consensus was that perhaps study lacked any obvious mechanism.

However in light of your query we have now consulted again and I'm pleased to say that we have reversed our original decision and would be happy to send the paper out for review.

I look forward to handling your manuscript for Development.

Resubmission

First decision letter

MS ID#: DEVELOP/2023/202333

MS TITLE: Disruption of maternal ACKR3 has profound effects on embryos and offspring.

AUTHORS: Ayumi Fukuoka, Gillian J Wilson, Elise Pitmon, Lily Koumbas-Foley, Hanna Johnsson, Marieke Pingen, and Gerard Graham

I have now (finally and with apologies for the delay) received all the referees' reports on the above manuscript, and have reached a decision. The referees' comments are appended below, or you can access them online: please go to BenchPress and click on the 'Manuscripts with Decisions' queue in the Author Area.

As you will see, the referees express considerable interest in your work, but have some significant criticisms and recommend a substantial revision of your manuscript before we can consider publication. If you are able to revise the manuscript along the lines suggested, which may involve further experiments, I will be happy receive a revised version of the manuscript. Your revised paper will be re-reviewed by one or more of the original referees, and acceptance of your manuscript will depend on your addressing satisfactorily the reviewers' major concerns. Please also note that Development will normally permit only one round of major revision. If it would be helpful, you are welcome to contact us to discuss your revision in greater detail. Please send us a point-by-point response indicating your plans for addressing the referees' comments, and we will look over this and provide further guidance.

Please attend to all of the reviewers' comments and ensure that you clearly highlight all changes made in the revised manuscript. Please avoid using 'Tracked changes' in Word files as these are lost in PDF conversion. I should be grateful if you would also provide a point-by-point response detailing how you have dealt with the points raised by the reviewers in the 'Response to Reviewers' box. If you do not agree with any of their criticisms or suggestions please explain clearly why this is so.

Reviewer 1

Advance summary and potential significance to field

The data show the compartimentalization of CXCL12 between maternal and embryonal circulation by syncytiotrophoblsts. This finding is new. Moreover, the authors convincingly show that ACKR3 inhibition leads to defective lymphopoiesis and hematopoiesis. As consequence micw born from mice treated with an established ACKR3 inhibitor show defective immune responses.

© 2024. Published by The Company of Biologists under the terms of the Creative Commons Attribution License (https://creativecommons.org/licenses/by/4.0/).

Comments for the author

In this study Fukuoka et al. intend to show that CXCL12 is compartmentalized between maternal and embryonal circulation. They identify syncytiotrophoblasts as ACKR3 expressing cells that can scavenge CXCL12 at the barrier tissue between maternal end embryonal circulation. Systemic blockade of ACKR3 with CCX771 leads to defective B-call hematopoiesis in embryos and results in higher susceptibility to inflammation in neonates.

The message is novel and interesting but not fully convincing.

Figure 1 (and supplementary) clearly shows that ACKR3 is expressed in the placenta of the mouse placenta starting at day 12.5 but is not expressed in hematopoietic (CD45+) and endothelial cells (CD31). The placenta does not necessarily stand for the embryo. The authors should also show the expression of ACKR3 in embryos. Deficiency of ACKR3 (like CXCL12 and CXCR4) in mice is lethal with strong defects seen in the pups including the CD31+ vascularization.

Figure 2 the message of the figure is obscured due to wrong assumptions. The datasheets of human DuoSet ELISA (DY350) and mouse DuoSet ELISA (DY450), both clearly state that the assays are cross-reacting with mouse and human CXCL12 respectively. They are not species-specific as stated. (Not surprising given the almost identical primary structure of CXCL12 from both species.). While this fact has little impact on the overall message, the data need to be interpreted differently.

The (low) levels of CXCL12 in ACKR3 ko pubs need explanation, because they are much lower than what is found in adults. Inhibition of ACKR3 in adults leads to an increase of normal serum CXCL12 levels from 0.5-2 ng/ml to 2-6 ng/ml (PubMed 24116850; 22110250; 34794236; 37554323). (The strong variation seen in Figure 2 may arise from platelet contamination). It would be important to measure CXCL12 expression in embryos by qPCR. Are the low levels due to scavenging of the chemokine by embryonal endothelial cells? Following injection of hCXCL12 maternal and embryonal plasma (serum) levels should be reported and compared.

The images (2E) need improvement (higher resolution). Are the red dots endosomal structures? Is it possible to measure CXCL12AF647 in embryos? (Is the raise shown in shown in 2D ACKR3+/- sufficient?). It may not be possible.

Figure 3 CCX771 passes the blood brain barrier (PubMed 21300915). It is surprising that the compound does not enter embryonal circulation. The lack of CCX771 passage to the embryo needs better documentation, particularly in the light of the perinatal death of ACKR3 deficient mice.

The expression of HSC and HPC in the presence of CCX771 could be discussed with respect to the immune cell development described by Sierro et.

In summary: the conclusion that trophoblasts are sufficient prevent entry of maternal CXCL12 in the embryonal circulation need to be corroborated. What are the levels of embryonal ACKR3 expression?

Minor:
Fig S3 The legends of panels A and B are inversed.

<u>Reviewer 2</u>

Advance summary and potential significance to field

The manuscript by Graham and colleagues investigates potential roles for the atypical receptor ACKR3 in regulating the transfer of maternal chemokines to developing embryos. The authors had termed this process ‘chemokine compartmentalisation’ in a previous study demonstrating that placental expression of the atypical receptor ACKR2 is required to prevent the transfer of inflammatory chemokines CCL2 and CCL7 into the circulation of the embryo. The current manuscript now prevents evidence that the scavenger receptor ACKR3 allows a similar

© 2024. Published by The Company of Biologists under the terms of the Creative Commons Attribution License (https://creativecommons.org/licenses/by/4.0/).

compartmentalisation of Cxcl12/CXCR4 signalling, a chemokine pathway that is widely studied for its roles in immune homing, cancer and development. This reviewer finds the proposal that scavenger receptors expressed in extraembryonic tissues help ‘isolate’ the developing fetus from fluctuations in maternal signals very interesting, especially for developmentally important chemokines like Cxcl12. It potentially offers an explanation for how immunological functions can continue in the mother without interfering in embryonic processes that are sensitive to chemokine levels, perhaps supporting the diversification of signaling functions.

Comments for the author

While interesting, the issue with the current manuscript is that it is very similar to the previous study, in question, approach and conceptual insights. In addition, it's unclear how appropriate this study is for Development, as there is little attempt to address developmental mechanisms or use methods that are typical of this field.

Major
1. Maternal vs Zygotic A basic problem I had with the current manuscript was distinguishing maternal functions from embryonic, as it appears to flip-flop between the two. This could be due to the authors considering everything outside the embryo proper as maternal, including extra-embryonic structures derived from the embryo. This issue could potentially be fixed with text changes but I’d like to resolve this first as it influences my recommendations for improvement on the experimental side. The study begins by showing expression of Ackr3 at the fetal maternal interface, more specifically, in syncytiotrophoblasts of the placenta. This non-specialist understands that these cells are derived from the embryonic trophoblasts, an impression that’s reinforced by the investigation into placental phenotypes in Ackr3 -/- embryos. They then show that Cxcl12 levels are increased in the plasma of Ackr3 homozygotes, as is the transfer of human Cxcl12 injected intravenously in pregnant mice (a nice experiment that they used for hCCL2 and hCCL7 in the previous study). Combined, these first data based on mutants establish a role for zygotic Ackr3 function in regulating levels of Cxcl12 in embryonic plasma.

Recommendation
The authors should make the text consistent with the standard definitions of maternal and embryonic/zygotic.
If the effects they are describing are due to the genotype of the embryo rather than the mother, these are zygotic. The ‘maternal ACKR3’ of the title works if it is only the maternal ACKR3 genotype that matters.

Alternatively, they can explain why they consider these placental defects as evidence for ‘maternal ACKR3’ functions, perhaps this is a distinction that is commonly used by specialists of mammalian development (?). I would agree that ‘maternal Cxcl12 chemokine’ appears relevant for the described phenotypes, but the ACKR3 receptor appears to be very much zygotic.

2. Placental specificity The latter experiments are presented as support for “roles for placental ACKR3 in limiting CXCL12 movement from maternal circulation to the embryo”. Given that ACKR3 is known to be expressed widely in embryos, it is not clear to me how they can be sure that the described changes in ACKR3 -/- are entirely down to loss of function in the placenta specifically, rather than a more widespread scavenging defect in deficient embryos.
Further experiments to examine the ‘developmental relevance of placental ACKR3’ involve injection of a pharmacological ACKR3 blocker (CCX771) into pregnant wild type mice, which leads to smaller, ‘pale-looking neonates’ when combined with co-injection of Cxcl12. CCX771 is not detectable in embryonic plasma, leading the authors to conclude that its effects are due to its action on placental ACKR3 (which they term maternal).
However, given that the impact of CCX771 on development is not well defined, I am not yet convinced its effects are specific to placental cells or even ACKR3 itself. This is a general issue with using small molecule inhibitors to define new developmental effects, thus such experiments are usually supported with genetic methods where gene and tissue specificity come baked-in. My guess is that the typical reader of Development will expect something along these lines.

© 2024. Published by The Company of Biologists under the terms of the Creative Commons Attribution License (https://creativecommons.org/licenses/by/4.0/).

Recommendation
The specific requirement for ACKR3 should be localized to the placenta using tissue-specific genetic methods. The testable prediction is that specific loss of ACKR3 function in the placenta will give rise to the described effects in embryos that are otherwise wild type. This will provide strong support for the exciting idea that it is specifically this population of scavenger receptors that buffer the embryo. Given that this function is apparently* derived from the embryonic trophectoderm (see point 1), this may not require tissue specific Cre drivers etc. Suggestion: Previous work from Nagy and others has shown that it is relatively simply to generate embryos where they extraembryonic tissues are of different genotype from the embryo (eg Tanaka M et al. Methods Mol Biol. 2001). In short, tetraploid embryos can be exploited to generate entirely ES derived mice that are supported by 'host' tetraploid trophectoderm (extraembryonic cells are less sensitive to aneuploidy). Thus, the authors could quickly generate embryos where only the placenta is deficient in ACKR3 and address resultant maternal Cxcl12 influx and phenotypes.

3. Specific impact of Cxcl12 transport An alternative way for the authors to extend beyond their previously published study would be to strengthen the link between the proposed influx of maternal Cxcl12 and the described phenotypes. Several pieces of data indicate that increased transport of Cxcl12 into the embryo is the cause of the described embryonic/b-cell/inflammatory phenotypes, with intravenous Cxcl12 injection enhancing the effect of CCX771 treatment.
However, these experiments are limited by the aforementioned specificity issues associated with the inhibitor (point 2).

Recommendation
Ideally, the authors could recapitulate these phenotypes by increasing the concentration of injected Cxcl12 alone, or in combination with genetic manipulation of ACKR3. From Fig 2 it appears that heterozygosity of ACKR3 (ACKR3/gfp) allows greater influx of injected Cxcl12/hCxcl12, suggesting that the placental ACKR3 gatekeeper could be 'overpowered'. This would strengthen the model.
Support from the specificity of the blocker could come from showing similar phenotypes in Ackr3 homozygous mutants. This again raises issue 1 ie whether the described phenotype is due to maternal or zygotic function. I can understand why the 'perinatal' lethality excludes addressing strictly maternal functions, as it is not possible to easily generate female adults. However, if I am correct in my assumption that the ACKR3 function is dependents on the zygotic genotype, and embryo-derived cells in the placenta then it should be possible to observe at least some of the same defects in homozygous neonates.

Minor
1. The study describes a number of effects of CCX771/Cxcl12 injection, with most insight arguably coming from their investigation into its impact on hematopoiesis/inflammation (arguably the best understood function for Cxcl12). Its embryonic growth phenotype is only superficially described, however this aspect is more prominent in the title/abstract, where it is described as "profound effects on embryos and offspring" (presumably to appeal to the target readership?). Given that Development routinely publishes papers describing embryos with altered body axes, missing organs, duplicated limbs etc, I doubt that the readership would agree that smaller, pale-looking neonates represents a profound effect on embryos. I'd recommend the authors focus these texts on the points where most insight is presented.

First revision

<u>Author response to reviewers' comments</u>

Reviewer 1 Comments for the author

The message is novel and interesting but not fully convincing.
Figure 1 (and supplementary) clearly shows that ACKR3 is expressed in the placenta of the mouse placenta starting at day 12.5 but is not expressed in hematopoietic (CD45+) and endothelial cells

© 2024. Published by The Company of Biologists under the terms of the Creative Commons Attribution License (https://creativecommons.org/licenses/by/4.0/).

(CD31). The placenta does not necessarily stand for the embryo. The authors should also show the expression of ACKR3 in embryos. Deficiency of ACKR3 (like CXCL12 and CXCR4) in mice is lethal with strong defects seen in the pups including the CD31+ vascularization.
We fully agree with the reviewer that the placenta does not represent the embryo and that there are alternative sites of ACKR3 expression in the embryo. We have now included data from a QPCR analysis of ACKR3 expression from E18.5 embryos which does indeed reveal expression in the embryo proper and also confirms that the placenta, at this time point is the major site of expression (supplementary Figure S1A and text on revised page 5). We hope this is of some use. We also hope that the data described below, from the Cyp19-Cre experiments (in response to comments from Reviewer 2), sufficiently persuades the reviewer that the phenotype relates specifically to trophoblast ACKR3 and not ACKR3 in other embryonic tissues.

Figure 2 the message of the figure is obscured due to wrong assumptions. The datasheets of human DuoSet ELISA (DY350) and mouse DuoSet ELISA (DY450), both clearly state that the assays are cross-reacting with mouse and human CXCL12, respectively. They are not species-specific as stated. (Not surprising given the almost identical primary structure of CXCL12 from both species.). While this fact has little impact on the overall message, the data need to be interpreted differently.
We fully agree and thank reviewer for pointing this error out. As the reviewer says, this does not change the overall message but we have now removed any wording referring to this as a human-specific CXCL12 ELISA. We apologise for this carelessness.

The (low) levels of CXCL12 in ACKR3 ko pups need explanation, because they are much lower than what is found in adults. Inhibition of ACKR3 in adults leads to an increase of normal serum CXCL12 levels from 0.5-2 ng/ml to 2-6 ng/ml (PubMed 24116850; 22110250; 34794236; 37554323). (The strong variation seen in Figure 2 may arise from platelet contamination). It would be important to measure CXCL12 expression in embryos by qPCR. Are the low levels due to scavenging of the chemokine by embryonal endothelial cells? Following injection of hCXCL12 maternal and embryonal plasma (serum) levels should be reported and compared.
We apologise if the way in which we have presented these data has caused some confusion. When collecting embryonic blood, we collect into PBS and it is therefore diluted. Accordingly, the data are expressed as pg/mg of total protein and not as pg/ml. In fact, when one takes into account a general murine plasma protein concentration of 80mg/ml, the values that we have come out very close to 2ng/ml increasing considerably to 5-30ng/ml in embryos from mothers injected with human CXCL12. We have inserted these calculations, and this logic, on page 6 of the revised manuscript.

The images (2E) need improvement (higher resolution). Are the red dots endosomal structures? Is it possible to measure CXCL12AF647 in embryos? (Is the raise shown in shown in 2D ACKR3+/- sufficient?). It may not be possible.
We apologise for the poor quality of the original image and have now improved this in the revised Figure 2E. We hope that this is now acceptable. In terms of whether the red dots are endosomes, or not, we are currently unclear but have included a short note on revised page 6 suggesting that they may well be endosomes. We hope that this is appropriate. Unfortunately, it is not possible to specifically measure CXCL12AF647.

Figure 3 CCX771 passes the blood brain barrier (PubMed 21300915). It is surprising that the compound does not enter embryonal circulation. The lack of CCX771 passage to the embryo needs better documentation, particularly in the light of the perinatal death of ACKR3 deficient mice.
We are not entirely clear why CCX771 passes through the blood-brain barrier but does not pass through the placenta. It may be due to the relatively indolent blood flow in the placenta compared to the blood-brain barrier. We would be happy to include a short sentence on this if that would be useful. That being said, we would point out that there is no correlation between the amount of CCX771 entering the embryonic circulation and any of the phenotypes observed. We have added short sentences, on revised Page 7 and 11, further highlighting this. In addition, the data from the Cyp19-Cre experiments described below in response to comments from Reviewer 2, hopefully will persuade the reviewer that the effects of CCX771 that we report can be explained on the basis of its impact on trophoblast, rather than intra-embryonic, ACKR3.

The expression of HSC and HPC in the presence of CCX771 could be discussed with respect to the immune cell development described by Sierro et.

© 2024. Published by The Company of Biologists under the terms of the Creative Commons Attribution License (https://creativecommons.org/licenses/by/4.0/).

We have now included a discussion of this point on revised page 10 and fully accept that we should have done this in the original version of the manuscript. We apologise for this.

Minor:
Fig S3 (new Fig S5) The legends of panels A and B are inversed.
We apologise for this carelessness and have now altered this figure legend.

Reviewer 2 Advance summary and potential significance to field

Reviewer 2 Comments for the author
While interesting, the issue with the current manuscript is that it is very similar to the previous study, in question, approach and conceptual insights. In addition, it's unclear how appropriate this study is for Development, as there is little attempt to address developmental mechanisms or use methods that are typical of this field.
The reviewer is right in that, mechanistically, this is similar to our previous study. However, we believe that the strength of the current manuscript lies in the considerably more profound phenotypes that we are reporting. In addition, the effects of interfering with ACKR3 are fundamentally different from those resulting from interference with ACKR2. As to relevance for Development, this really hinges on the importance of CXCL12 for stem cell development and the fact that the effects of interfering with ACKR3 are established within the embryo. We hope that the reviewer agrees that the strength of the phenotype that we report, and the relevance to embryogenesis, does indeed make this manuscript relevant for Development.

Major

1. Maternal vs Zygotic
A basic problem I had with the current manuscript was distinguishing maternal functions from embryonic, as it appears to flip-flop between the two. This could be due to the authors considering everything outside the embryo proper as maternal, including extra-embryonic structures derived from the embryo. This issue could potentially be fixed with text changes but I'd like to resolve this first as it influences my recommendations for improvement on the experimental side. The study begins by showing expression of Ackr3 at the fetal maternal interface, more specifically, in syncytiotrophoblasts of the placenta. This non-specialist understands that these cells are derived from the embryonic trophoblasts, an impression that's reinforced by the investigation into placental phenotypes in Ackr3 -/- embryos. They then show that Cxcl12 levels are increased in the plasma of Ackr3 homozygotes, as is the transfer of human Cxcl12 injected intravenously in pregnant mice (a nice experiment that they used for hCCL2 and hCCL7 in the previous study). Combined, these first data based on mutants establish a role for zygotic Ackr3 function in regulating levels of Cxcl12 in embryonic plasma.

Recommendation
The authors should make the text consistent with the standard definitions of maternal and embryonic/zygotic.
If the effects they are describing are due to the genotype of the embryo rather than the mother, these are zygotic. The 'maternal ACKR3' of the title works if it is only the maternal ACKR3 genotype that matters. Alternatively, they can explain why they consider these placental defects as evidence for 'maternal ACKR3' functions, perhaps this is a distinction that is commonly used by specialists of mammalian development (?). I would agree that 'maternal Cxcl12 chemokine' appears relevant for the described phenotypes, but the ACKR3 receptor appears to be very much zygotic.

We completely agree with these comments and much of this confusion is a result of some very poor wording in our original manuscript and our clumsy attempts to communicate the fact that the inhibition was the result of maternal administration of CCX771. In particular, our title was extremely misleading, and we apologise for this. In fact, we are quite clear that the effects that we are seeing are trophoblast-specific and therefore clearly embryonic/zygotic and not maternal. We have changed the title of the paper and have changed the wording at appropriate places to be much clearer on this point (Pages 4 and 6 and we have replaced the word 'maternal' with 'trophoblast' at various points throughout the text). We hope that this is now acceptable to the reviewer.

© 2024. Published by The Company of Biologists under the terms of the Creative Commons Attribution License (https://creativecommons.org/licenses/by/4.0/).

2. Placental specificity
The latter experiments are presented as support for “roles for placental ACKR3 in limiting CXCL12 movement from maternal circulation to the embryo”. Given that ACKR3 is known to be expressed widely in embryos, it is not clear to me how they can be sure that the described changes in ACKR3 -/- are entirely down to loss of function in the placenta specifically, rather than a more widespread scavenging defect in deficient embryos.

Further experiments to examine the ‘developmental relevance of placental ACKR3’ involve injection of a pharmacological ACKR3 blocker (CCX771) into pregnant wild type mice, which leads to smaller, ‘pale-looking neonates’ when combined with co-injection of Cxcl12. CCX771 is not detectable in embryonic plasma, leading the authors to conclude that its effects are due to its action on placental ACKR3 (which they term maternal).
However, given that the impact of CCX771 on development is not well defined, I am not yet convinced its effects are specific to placental cells or even ACKR3 itself. This is a general issue with using small molecule inhibitors to define new developmental effects, thus such experiments are usually supported with genetic methods where gene and tissue specificity come baked-in. My guess is that the typical reader of Development will expect something along these lines.
Recommendation
The specific requirement for ACKR3 should be localized to the placenta using tissue-specific genetic methods. The testable prediction is that specific loss of ACKR3 function in the placenta will give rise to the described effects in embryos that are otherwise wild type. This will provide strong support for the exciting idea that it is specifically this population of scavenger receptors that buffer the embryo. Given that this function is apparently* derived from the embryonic trophectoderm (see point 1), this may not require tissue specific Cre drivers etc. Suggestion: Previous work from Nagy and others has shown that it is relatively simply to generate embryos where they extraembryonic tissues are of different genotype from the embryo (eg Tanaka M et al. Methods Mol Biol. 2001). In short, tetraploid embryos can be exploited to generate entirely ES derived mice that are supported by ‘host’ tetraploid trophectoderm (extraembryonic cells are less sensitive to aneuploidy). Thus, the authors could quickly generate embryos where only the placenta is deficient in ACKR3 and address resultant maternal Cxcl12 influx and phenotypes.

We have now included data from experiments using trophoblast-specific Cre (Cyp19-Cre) mice crossed with ‘floxed’ ACKR3 mice. These data confirm the central role for trophoblast ACKR3 in the phenotypes reported. The reasons that we did not use this approach to study is that this Cre-driver is associated with mosaic expression and unpredictable connectivity (see Page 4 of the revised manuscript). It is indeed possible to get data using this Cre approach but involves the use of numerous mice therefore raising ethical issues. The fact that the phenotypes observed from the Cre approach mirror those seen in the CCX771-treated mice gave us confidence that this inhibitor was faithful inhibiting trophoblast ACKR3. The data obtained from the Cre mouse crosses are described on pages 4, 6, 7, 8 and 10 of the revised manuscript with additional information on the mice provided in the Materials and methods section. The actual data are presented in Figures S3, S4 and S6. We hope that these new data adequately deal with the reviewer’s concerns.

3. Specific impact of Cxcl12 transport
An alternative way for the authors to extend beyond their previously published study would be to strengthen the link between the proposed influx of maternal Cxcl12 and the described phenotypes. Several pieces of data indicate that increased transport of Cxcl12 into the embryo is the cause of the described embryonic/b-cell/inflammatory phenotypes, with intravenous Cxcl12 injection enhancing the effect of CCX771 treatment. However, these experiments are limited by the aforementioned specificity issues associated with the inhibitor (point 2).

Recommendation
Ideally, the authors could recapitulate these phenotypes by increasing the concentration of injected Cxcl12 alone, or in combination with genetic manipulation of ACKR3. From Fig 2 it appears that heterozygosity of ACKR3 (ACKR3/gfp) allows greater influx of injected Cxcl12/hCxcl12, suggesting that the placental ACKR3 gatekeeper could be ‘overpowered’. This would strengthen the model.

Support from the specificity of the blocker could come from showing similar phenotypes in Ackr3 homozygous mutants. This again raises issue 1 ie whether the described phenotype is due to

© 2024. Published by The Company of Biologists under the terms of the Creative Commons Attribution License (https://creativecommons.org/licenses/by/4.0/).

maternal or zygotic function. I can understand why the 'perinatal' lethality excludes addressing strictly maternal functions, as it is not possible to easily generate female adults. However, if I am correct in my assumption that the ACKR3 function is dependents on the zygotic genotype, and embryo-derived cells in the placenta, then it should be possible to observe at least some of the same defects in homozygous neonates.
We hope that the additional data from the Cre mouse approach persuades the reviewer that the effects are specific to trophoblast ACKR3 and not, therefore, a consequence of off target effects of the blocker. As discussed above it is quite clear in our mind, and we hope that we have now made this more obvious in the revised text, that the effects are embryonic/zygotic.

Minor
1. The study describes a number of effects of CCX771/Cxcl12 injection, with most insight arguably coming from their investigation into its impact on hematopoiesis/inflammation (arguably the best understood function for Cxcl12). Its embryonic growth phenotype is only superficially described, however this aspect is more prominent in the title/abstract, where it is described as “profound effects on embryos and offspring” (presumably to appeal to the target readership?). Given that Development routinely publishes papers describing embryos with altered body axes, missing organs, duplicated limbs etc, I doubt that the readership would agree that smaller, pale-looking neonates represents a profound effect on embryos. I'd recommend the authors focus these texts on the points where most insight is presented.

We agree with this and have again altered the title to more accurate reflect what we report in the text. We hope that this adequately deals with the reviewer’s concern.

Second decision letter

MS ID#: DEVELOP/2023/202333

MS TITLE: Disruption of placental ACKR3 effects embryo, and offspring, growth and hematopoietic development.

AUTHORS: Ayumi Fukuoka, Gillian J Wilson, Elise Pitmon, Lily Koumbas-Foley, Hanna Johnsson, Marieke Pingen, and Gerard Graham

I have now received all the referees reports on the above manuscript, and have reached a decision. The referees' comments are appended below, or you can access them online: please go to BenchPress and click on the 'Manuscripts with Decisions' queue in the Author Area.

The overall evaluation is positive and we would like to publish a revised manuscript in Development. However, Reviewer 2 has a suggestion for moving one of the Supp Figures into the main manuscript. You might want to consider this suggestion and make this change in the final version of your paper.

Please attend to all of the reviewers' comments in your revised manuscript and detail them in your point-by-point response. If you do not agree with any of their criticisms or suggestions explain clearly why this is so. If it would be helpful, you are welcome to contact us to discuss your revision in greater detail. Please send us a point-by-point response indicating your plans for addressing the referees’ comments, and we will look over this and provide further guidance.

Reviewer 1

Advance summary and potential significance to field

In this study Fukuoka et al. show that CXCL12 is compartmentalized between maternal and embryonal circulation. They identify syncytiotrophoblasts as ACKR3 expressing cells that can scavenge CXCL12 at the barrier tissue between maternal end embryonal circulation. Systemic

© 2024. Published by The Company of Biologists under the terms of the Creative Commons Attribution License (https://creativecommons.org/licenses/by/4.0/).

blockade of ACKR3 with CCX771 leads to defective B-call hematopoiesis in embryos and results in higher susceptibility to inflammation in neonates.

Comments for the author

The authores have considered my comments to the first submision. The changes made fully answer my concerns. I hova no further comments.

Reviewer 2

Advance summary and potential significance to field

The authors show that the expression of the atypical chemokine receptor Ackr3, a scavenger of Cxcl12, in trophoectoderm cells protects the developing embryo from exposure to increased chemokine levels from the mother. When Ackr3 function is perturbed using a specific inhibitor or by cre-induced deletion in the trophectoderm, the offspring have a number of defects consistent with overexposure to Cxcl12.

Comments for the author

The authors have done a good job of addressing my previous concerns in the resubmitted manuscript. I appreciate their efforts to correct the confusing nomenclature and to shift the emphasis from the 'profound effect on embryos' to the more convincing findings (although I still find the title very chewy and confusing - "...embryo, and offspring, growth hematopoietic development"…is is the growth of both embryo and 'offspring'?).

This reviewer particularly appreciates that they now address the previous 'elephant in the room', namely, they restrict the loss of Ackr3 to placental cells using a trophoblast specific cre line (Cyp19-cre). While the effects are on the weaker side, when compared to administration of the inhibitor + cxcl12 (which they argue is due to the mosaicism of this line), these data provide important genetic support for their proposal that placental ackr3 protects the developing embryo from chemokine fluctuations.In fact, my only recommendation now would be to reposition the data shown in Fig S4 b.d to the main figures, ideally with a schematic describing the rationale (ie mosaic loss of the receptor in the trophoectoderm). I guess that many Development readers will expect such an approach to be applied.

Second revision

Author response to reviewers' comments

Please find files relating to our manuscript which has recently received a positive evaluation. We have revised the manuscript to move Figures S4b and S4d to the main figures and these are now presented as Figure 3e and 3f. Appropriate changes have also been made to the text. The reviewer also mentions ideally including a schematic describing the rationale concerning the mosaic loss of ACKR3 in the trophectoderm. We have not included this as hopefully the description of the mosaicism of the Cre-driver elsewhere in the text will fully serve this purpose. Please do let me know if you would still like us to insert some text here and we will be happy to do so.

Third decision letter

MS ID#: DEVELOP/2023/202333

MS TITLE: Disruption of placental ACKR3 effects embryo, and offspring, growth and hematopoietic development.

© 2024. Published by The Company of Biologists under the terms of the Creative Commons Attribution License (https://creativecommons.org/licenses/by/4.0/).

AUTHORS: Ayumi Fukuoka, Gillian J Wilson, Elise Pitmon, Lily Koumbas-Foley, Hanna Johnsson, Marieke Pingen, and Gerard Graham
ARTICLE TYPE: Research Article

I am happy to tell you that your manuscript has been accepted for publication in Development, pending our standard ethics checks.

© 2024. Published by The Company of Biologists under the terms of the Creative Commons Attribution License (https://creativecommons.org/licenses/by/4.0/).
